# Alcohol and nicotine codependence-associated DNA methylation changes in promoter regions of addiction-related genes

**DOI:** 10.1038/srep41816

**Published:** 2017-02-06

**Authors:** Hongqin Xu, Fan Wang, Henry R. Kranzler, Joel Gelernter, Huiping Zhang

**Affiliations:** 1 Department of Psychiatry, Yale University School of Medicine, New Haven, CT, USA; 2 Department of Psychiatry, University of Pennsylvania Perelman School of Medicine and VISN4 MIRECC, Crescenz VAMC, Philadelphia, PA, USA; 3 Department of Genetics, Yale University School of Medicine, New Haven, CT, USA; 4 Department of Neurobiology, Yale University School of Medicine, New Haven, CT, USA; 5 VA Connecticut Healthcare System, West Haven, CT, USA.

## Abstract

Altered DNA methylation in addiction-related genes may modify the susceptibility to alcohol or drug dependence (AD or ND). We profiled peripheral blood DNA methylation levels of 384 CpGs in promoter regions of 82 addiction-related genes in 256 African Americans (AAs) (117 cases with AD-ND codependence and 139 controls) and 196 European Americans (103 cases with AD-ND codependence and 93 controls) using Illumina’s GoldenGate DNA methylation array assays. AD-ND codependence-associated DNA methylation changes were analyzed using linear mixed-effects models with consideration of batch effects and covariates age, sex, and ancestry proportions. Seventy CpGs (in 41 genes) showed nominally significant associations (*P* < 0.05) with AD-ND codependence in both AAs and EAs. One CpG (*HTR2B* cg27531267) was hypomethylated in AA cases (*P* = 7.2 × 10^−5^), while 17 CpGs in 16 genes (including *HTR2B* cg27531267) were hypermethylated in EA cases (5.6 × 10^−9^ ≤ *P* ≤ 9.5 × 10^−5^). Nevertheless, 13 single nucleotide polymorphisms (SNPs) nearby *HTR2B* cg27531267 and the interaction of these SNPs and cg27531267 did not show significant effects on AD-ND codependence in either AAs or EAs. Our study demonstrated that DNA methylation changes in addiction-related genes could be potential biomarkers for AD-ND co-dependence. Future studies need to explore whether DNA methylation alterations influence the risk of AD-ND codependence or the other way around.

Alcohol and nicotine are the two most commonly used substances of abuse. In 2013, almost 17 million American adults had alcohol abuse or dependence^1^, and more than 42 million American adults were smokers^2^. Tobacco use and excessive alcohol consumption can result in substantial health problems. Tobacco use is the leading cause and excessive alcohol consumption is the third leading cause of preventable death in the United States, with more than 480,000 and 80,000 deaths, respectively, attributable to these behaviors in a one-year period^2^,^3^,^4^. Although alcohol and nicotine addiction are often viewed as separate disorders, they frequently co-occur. Individuals with alcohol dependence (AD) are four times more likely to be affected with nicotine dependence (ND), and nearly 23% of ND subjects met past-year criteria for alcohol use disorders^5^,^6^,^7^. Additionally, individuals with comorbid AD and ND had worse clinical outcomes than those with either AD or ND alone^8^.

AD and ND are both genetically-influenced complex disorders with both shared and specific genetic risk factors. The heritability of AD is estimated to be 50–70%^9^,^10^,^11^,^12^,^13^ and that of ND is estimated to be 40–60%^11^,^14^. The co-occurrence of AD and ND suggests a substantial genetic correlation between these two disorders and a possible common genetic vulnerability. The common genetic influence may partially explain the observations that smoking was a significant risk factor for promoting the progression of AD^15^,^16^, while AD was associated with greater nicotine withdrawal^17^. The reinforcing effects of alcohol and nicotine could be mediated by common reward pathways^18^,^19^. Genetic and epigenetic variants in genes involved in these reward pathways may confer vulnerability to AD-ND codependence.

Genome-wide association studies (GWAS) have been performed to identify genetic variants that are associated with AD and/or ND. At least eight original GWAS on AD have been conducted^20^,^21^,^22^,^23^,^24^,^25^,^26^,^27^. Findings from these AD GWAS were largely inconsistent, with the exception of variants in genes encoding the alcohol-metabolizing enzymes^28^. At least nine original GWAS on ND have been published^29^,^30^,^31^,^32^,^33^,^34^,^35^,^36^,^37^; three of them reported genome-wide significant results for single nucleotide polymorphisms (SNPs) in exon 5 of *CHRNA3* on chromosome 15^32^, the upstream region of *IL15* on chromosome 4^33^, the intronic region of *DLC1* on chromosome 8^37^, or intergenic regions on chromosomes 7 (between *CACNA2D1* and *PCLO*), 8 (flanked by *INTS10*), or 14 (from 45,307,535 to 45,613,093 base pairs or from rs146754986 to rs145624594)^37^. To increase the sample size and statistical power, several meta-analyses of GWAS on AD or ND have been conducted. For example, genome-wide meta-analyses identified a locus [located in the intronic region of the autism susceptibility candidate 2 gene (*AUTS2*)] that was associated with alcohol consumption and multiple loci (including *CHRNA3* rs1051730) that were associated with smoking behaviors^38^. To date, no studies are known to have studied the pleiotropic effects of gene variants on both AD and ND. Only two published GWASs have studied the association of genetic variants with the co-occurrence of AD and ND (or AD-ND codependence): one identified genome-wide significant SNPs that are located near *MARK1* on chromosome 1, proximal to *DDX6* on chromosome 11, or in the intronic region of *KIAA1409* on chromosome 14^39^, and another, based exclusively on publicly-available data, found genome-wide significant association signals between *IPO11* and *HTR1A* on chromosome 5^40^.

Although a number of AD and/or ND-associated genetic variants have been identified by GWAS or candidate gene studies, they explain only a small proportion of the genetic variance for these disorders^38^,^41^. Among the likely explanations for the “missing” genetic variance are epigenetic events (such as DNA methylation, histone modifications, chromatin remodeling, and noncoding RNA regulation), rare genetic variation, copy number variants, and the interaction among genes and between genes and environment. There is also evidence that environmental factors exert their effects on gene transcription through epigenetic mechanisms^42^. Candidate gene DNA methylation or epigenome-wide association studies (EWAS)^43^,^44^,^45^,^46^,^47^,^48^, including ours^49^,^50^, have shown altered DNA methylation in the peripheral blood of AD subjects or lymphoblastoid cell lines derived from AD subjects. We^51^ and others^52^,^53^ have also found altered DNA methylation in postmortem brains of AD subjects. Similarly, ND-associated DNA methylation changes have been found in the peripheral blood/lymphoblast cell lines^46^,^54^,^55^, lung^56^, or other tissues^57^. We are not aware of any published studies that have examined epigenetic changes contributing to AD-ND codependence. Given that AD and ND are genetically influenced complex disorders that exhibit a high degree of comorbidity, we examined AD-ND codependence-associated DNA methylation alterations in the promoter regions of 82 addiction-related genes in two populations: African Americans (AAs) and European Americans (EAs).

## Results

### AD-ND codependence-associated promoter DNA methylation changes in addiction-related genes

**Figure 1:**
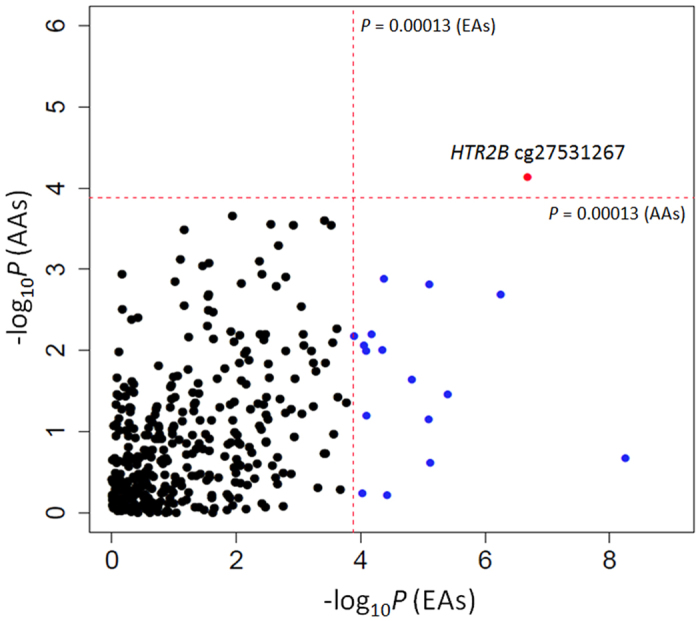
Distribution of *P* values of 384 CpGs in 82 addiction-related genes in African Americans and European Americans. The association between 384 CpGs in 82 addiction-related genes and alcohol and nicotine codependence was analyzed using the liner mixed-effect model. There were 17 CpGs survived the Bonferroni adjustment (*P* < = 0.05/384 = 0.00013; the red dashed-line) in European Americans. Only one CpGs (cg27531267 in the promoter region of *HTR2B*) survived the Bonferroni correction in both African Americans (AAs) and European Americans (EAs).

Methylation levels of 384 CpGs in the promoter region of 82 addiction-related genes were examined by the custom-designed Illumina GoldenGate assay for DNA methylation profiling, and AD-ND codependence-associated DNA methylation changes were identified in both AAs and EAs. The association analysis results are summarized in Supplementary Tables S1 and S2. In AAs, 103 (26.8%) CpGs in 54 genes showed nominally significant associations (*P* < 0.05) with AD-ND codependence (Supplementary Table S1). Only one CpG (cg27531267, located in the promoter region of *HTR2B*) remained significant (*P* = 7.2 × 10^−5^) after Bonferroni correction for multiple testing (*P* value ≤ 0.05/384 = 1.3 × 10^−4^ as the corrected statistical significance threshold), and it was hypomethylated in AAs with AD-ND codependence (Table 1). In EAs, 152 (39.6%) CpGs in 63 genes showed nominally significant associations (*P* < 0.05) with AD-ND codependence (Supplementary Table S2). The findings from 17 CpGs in 16 genes (*MBD3* cg21372728, *HTR2B* cg27531267, *PENK* cg26106216, *DRD2* cg05421426, *NCAM1* cg14313206, *NCAM1* cg21572351, *HTR2C* cg02156408, *HTR3A* cg08989585, *GABRB2* cg02095443, *OPRD1* cg01706569, *DRD4* cg08079114, *RGS17* cg12505522, *SLC6A4* cg14534584, *SLC6A3* cg00037218, *OPRK1* cg07344165, *GAD1* cg04123893, and *GABRB1* cg21074850) survived Bonferroni correction (5.6 × 10^−9^ ≤ *P* ≤ 9.5 × 10^−5^, using *P* value ≤ 0.05/384 = 1.3 × 10^−4^ as the statistical significance threshold). All 17 CpGs were hypermethylated in EAs with AD-ND codependence (Table 1). As displayed in Fig. 1, the findings from *HTR2B* cg27531267 withstood multiple testing corrections in both AAs (AAs: *P* = 7.2 × 10^−5^) and EAs (*P* = 2.1 × 10^−7^). Additionally, 70 CpGs (in 41 genes) showed nominally significant associations (*P* < 0.05) with AD-ND codependence in both AAs and EAs (Supplementary Tables S1 and S2).

### No significant interactive effects of HTR2B cg27531267 and nearby SNPs on AD-ND codependence

Considering that genetic variants may either have a direct or an indirect (e.g., *via* altering DNA methylation patterns) influence on disease risk, we further analyzed the effects of 13 SNPs around *HTR2B* cg27531267 (50 kb up- or downstream of cg27531267) and the interaction of these SNPs with *HTR2B* cg27531267 on the susceptibility to AD-ND codependence. All 13 SNPs were located in a tight linkage disequilibrium (LD) block with average R2 of 0.48 in EAs, while the LD pattern was similar but less tight in AAs with average R2 of 0.28 (Figure S1). As shown in Table 2, none of the 13 SNPs was significantly associated with AD-ND codependence in either AAs or EAs. Moreover, the interactive effect of *HTR2B* cg27531267 and nearby SNPs on AD-ND codependence was not significant in either AAs or EAs. Additionally, the methylation level of *HTR2B* cg27531267 was not significantly correlated with the genotypes of 13 nearby SNPs in either AAs or EAs (*P*_correlation_ > 0.05).

### Potential function of AD-ND codependence-associated CpGs

**Figure 2:**
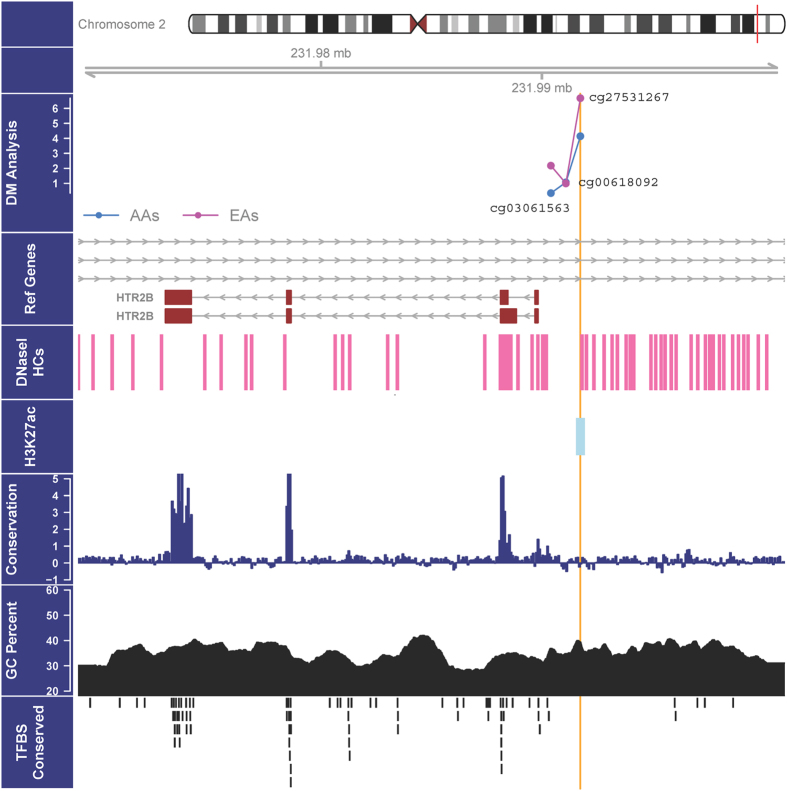
Functional annotation of three *HTR2B* promoter CpGs. The top panel contains the -log *P* values for the association between DNA methylation of three *HTR2B* promoter CpGs and alcohol-nicotine codependence. The other panels indicate the presence of coding exons (boxes in dark red) and noncoding introns (grey lines) of *HTR2B* (the second panel), DNase hypersensitivity cluster sites (the third panel), the location of a H3K27ac histone modification (the fourth panel), the conservative area (the fifth panel), the percentage of G (guanine) and C (cytosine) bases (the 6th panel), and the location of CpGs in transcription factor binding sites (TFBSs) (the seventh panel). The function of the most significant CpG (i.e., cg27531267 in the promoter region of *HTR2B*) is annotated by a brown line across all panels.

The program PROMO was used to predict putative transcription factor binding sites (TFBS) in DNA sequences harboring differentially methylated CpGs. Among the 17 differentially methylated CpGs (Table 1), 11 were predicted to be located in the core binding site of one or more transcription factors (Table 3). The UCSC Genome Browser was used to query DNase hypersensitivity sites (DHSs) and H3K27Ac marks in DNA sequences harboring differentially methylated CpGs. Among the 17 differentially methylated CpGs, 14 were located in DHSs and five were situated in DNA sequences that are associated with the H3K27Ac histone mark (Table 3). Although the most significant *HTR2B* cg27531267 was not predicted to be located in TFBSs, it was mapped to DHSs and DNA sequences associated with histone protein modification mark H3K27Ac (Fig. 2).

## Discussion

Prior evidence supports the involvement of many of the genes included in the present study in neurobiologic processes underlying drug reward and addiction. However, with a few exceptions (such as those coding for nicotinic acetylcholine receptors or alcohol metabolizing enzymes), most of these genes have not been previously found to harbor genetic variants that are associated at a genome-wide level with AD and/or ND. There are other mechanisms by which these loci could exert more substantial effects on AD and/or ND: namely, DNA methylation changes in addiction-related genes could confer vulnerability to AD and/or ND. In the present study, we identified methylation alterations in the promoter regions of a number of addiction-related genes in African Americans (AAs) and European Americans (EAs) with AD-ND codependence.

We found that differentially methylated genes are involved in several critical pathways for AD and/or ND. A *post hoc* power analysis demonstrated that 196 EAs samples provided 80% statistical power for an effect size greater than 0.004, while 256 AA samples provided 80% statistical power to detect an effect size greater than 0.003, indicating that our sample size provided adequate statistical power to detect methylation changes. As summarized in Table 1, four of the differentially methylated genes were serotonergic (*HTR2B, HTR2C, HTR3A*, and *SLC6A4*), three were dopaminergic (*DRD2, DRD4*, and *SLC6A3*), two were GABAergic (*GABRB1* and *GABRB2*), one was glutamatergic (*GAD1*), and three (*OPRD1, OPRK1*, and *PENK*) were opioidergic. Three other genes (*RGS17, NCAM1*, and *MBD3*), which do not belong to the above-listed neurotransmitter systems, also showed promoter DNA methylation changes in subjects with AD-ND codependence. *RGS17* encodes a regulator of G-protein signaling (RGS)^58^, which can inactivate the G protein and rapidly switch off G-protein-coupled receptor signaling pathways^59^. Our candidate gene studies have demonstrated that variation in *RGS17* is associated with a variety of different substance dependence disorders^60^. *NCAM1* encodes a neural cell adhesion protein, a member of the immunoglobulin superfamily^61^. It is located in the *DRD2*-*ANKK1*-*TTC12*-*NCAM1* gene cluster region, and SNPs or haplotypic variants in this region were found to be associated with dependence on alcohol, nicotine, or other drugs of abuse^62^,^63^,^64^. *MBD3* encodes methyl-CpG binding domain protein 3, a nuclear protein that is potentially involved in chromatin remodeling and histone modifications^65^,^66^. Because genetic association studies did not reveal a strong effect (i.e., with genome-wide significance) of variants in the above genes on AD and/or ND, we postulate that either inherent DNA methylation of these genes results in AD and/or ND or long-term alcohol misuse or smoking leads to AD and/or ND through epigenetic modifications. It should be noted that these epigenetic changes may not contribute to the risk of AD and ND simultaneously or to the same extent. Significant CpGs identified in the present study are associated with AD-ND codependence, which was considered to be a new phenotype, *i.e.*, the co-occurring risk of AD and ND.

Our findings suggest that the impact of DNA methylation of addiction-related genes on AD-ND codependence risk may be larger than that of genetic variants carried by these genes. Considering the possible correlation of methylation levels of CpGs and genotypes of nearby SNPs as reported in our previous studies^67^, we further investigated the effect of 13 SNPs within ± 50 kb of *HTR2B* cg27531267, as well as the interaction of cg27531267 with these SNPs on AD-ND codependence risk. Although *HTR2B* cg27531267 was differentially methylated in both AAs and EAs (albeit in opposite directions) with AD-ND codependence, no interactive effect of *HTR2B* cg27531267 with proximal SNPs on AD-ND codependence was observed in either population. Additionally, none of the 13 nearby SNPs was significantly associated with AD-ND codependence (Table 2). This finding is consistent with the results from our previous GWAS research that variants in *HTR2B* were not significantly associated with either AD^26^ or ND^37^.

The present study provides further evidence that DNA methylation changes within the regulatory (promoter) regions of addiction-related genes are associated with AD and/or ND, presumably because they may result in altered gene transcription. The rationale is that promoter DNA methylation may directly interfere with the binding of transcription factors (TFs) to the regulatory regions. Among the top 17 CpGs located in promoter regions of 16 genes (Table 1), 11 were predicted to be located in the core binding site of one or more TFs (Table 3). It is well known that chromatin structure mediates the interaction of TFs and DNA^68^,^69^. Our findings suggest that promoter DNA methylation may also modulate TF-DNA interactions and subsequently influence gene transcription. Moreover, methylated CpG falling within DNase hypersensitivity sites (DHSs) may impede the association of TFs to DNA, thus inhibiting the accessibility of chromatin. Because we did not extract RNA from blood samples (which were used only for genomic DNA extraction), we were unable to perform RT-qPCR to confirm gene expression changes caused by differentially methylated CpGs. Additionally, methylation of CpGs mapped to DNA sequences that are associated with histone marks (e.g., H3K27Ac mark, which is often found near active regulatory elements) may change histone protein epigenetic status, thus influencing the compact structure of chromatins. We also noticed that some of the 17 differentially methylated CpGs (including the most significant *HTR2B* cg27531267) were located in DHSs or DNA sequences that are associated with histone mark H3K27Ac. Taken together, subjects with altered DNA methylation in promoter regions of addiction-related genes may have an increased risk of AD and/or ND. Note that, further studies are needed to replicate the above findings in independent samples and extend the findings to other racial/ethnic groups.

The major limitation of the present study is that we only investigated the association of CpGs in promoter regions of a number of preselected addiction-related genes. For a more complete understanding of the epigenetic mechanism of AD-ND codependence, it will be necessary to use a high-resolution DNA methylation array (such as the Illumina MethylationEPIC BeadChip) assays or whole genome bisulfite sequencing (WGBS) to identify DNA methylomic changes. Another limitation is that we did not consider the relative proportion of different types of blood cells in the DNA methylation data analysis. Inter-individual differences in DNA methylation levels due to different blood cell composition may confound the findings. In future, when we have the high-density DNA methylation data (such as those generated by the Illumina MethylationEPIC BeadChip), we could use the method developed by Jaffe and Irizarry^70^ to estimate the relative proportions of CD4^+^ and CD8^+^ T-cells, natural killer cells, monocytes, granulocytes, and B-cells in blood samples and then incorporate the cell proportion estimation into the data analysis. Although DNA methylation patterns in the peripheral blood may not reflect those in the brain, peripheral blood samples are easier to collect than brain tissue samples, and blood DNA methylation changes in regulatory regions of genes could be accessible biomarkers.

We are aware that there are more significant CpGs in EAs than AAs, even though the EA sample was smaller than the AA sample. Additionally, it is unknown why *HTR2B* cg27531267 showed an opposite methylation direction in AAs (hypomethylation) and EAs (hypermethylation) with AD-ND codependence. One possible explanation is that the DNA methylation status of CpGs can be influenced by genetic variation. Similar to many disease-associated SNPs that are specific to a certain population, CpG methylation levels may also be population-specific, leading to inconsistent results between EAs and AAs. For example, the top CpG cg27531267 showed a mean methylation level of 0.045 in AA controls, which was significantly higher than that of EA controls (β = 0.035, *P* = 1.8 × 10^−8^). Among the 13 SNPs that are proximal to *HTR2B* cg27531267 and included in CpG-SNP interaction analysis, two were either not existent or rare in EAs (Table 2).

In summary, the present study examined DNA methylation alterations in promoter regions of addiction-related genes among individuals with AD-ND codependence. We identified both specific and shared DNA methylation changes in the two populations. The overlap of the differentially methylated promoter CpGs and TFBSs or DHSs and the location of differentially methylated CpGs in DNA sequences that are associated with specific histone marks (e.g., H3K27Ac) imply that promoter CpG methylation may modulate gene transcription and influence an individual’s susceptibility to AD-ND codependence. Considering the reversibility of DNA methylation, the findings from the present study could provide the basis for effective pharmacotherapies for AD-ND co-dependence that target specific epigenetic marks in promoter regions of addiction-related genes.

## Methods

### Subjects

Two hundred fifty-six African Americans (AAs) [including 117 cases (71 males and 46 females) with AD-ND codependence and 139 controls (31 males and 108 females)] and 196 European Americans [including 103 cases (59 males and 44 females) with AD-ND codependence and 93 controls (49 males and 44 females)] were recruited from substance abuse treatment centers or through advertisements at the University of Connecticut Health Center (n = 200), Yale University (n = 126), and the Medical University of South Carolina (n = 126). The mean age of AA cases and AA controls was 42 ± 8 and 37 ± 14 years, respectively (*P* < 0.05). The mean age of EA cases and EA controls was 43 ± 12 and 37 ± 16 years, respectively (*P* > 0.05). Both cases and controls were chosen from a large sample of subjects recruited for studies of the genetics of substance dependence. Subjects were interviewed using an electronic version of the Semi-Structured Assessment for Drug Dependence and Alcoholism (SSADDA)^71^. Lifetime diagnoses for AD and ND codependence were made according to the criteria of the Diagnostic and Statistical Manual of Mental Disorders, 4th edition (DSM-IV) [American Psychiatric Association, 1994]. None of the subjects had a lifetime major psychotic disorder such as schizophrenia and bipolar disorder. Additionally, no control subjects (139 AAs and 93 EAs) were affected with alcohol or drug abuse or dependence. Subjects gave informed consent as approved by the institutional review board at each clinical site, and certificates of confidentiality were obtained from the National Institute on Drug Abuse and the National Institute on Alcohol Abuse and Alcoholism. All methods were carried out in accordance with relevant guidelines and regulations. All experimental protocols were approved by the Human Investigation Committee of the above three institutes.

The self-reported genetic background of all subjects included in the present study was verified using a set of 41 ancestry informative markers (AIMs), including 36 short tandem repeat markers and five SNPs, implemented in the program STRUCTURE^72^. Subjects were defined as EAs if their ancestry proportion scores were less than 0.5; otherwise, they were considered AAs. To minimize the influence of subjects’ genetic background on the results of the DNA methylation analysis, subjects’ ancestry proportions were considered as a covariate in the differential CpG analysis.

### DNA extraction and bisulfite modification

Genomic DNA was extracted from the peripheral blood of cases and controls using the PAXgene Blood DNA Kit (PreAnalytiX, Hombrechtikon, Switzerland). One microgram of genomic DNA was treated with the bisulfite reagent included in the EZ DNA Methylation Kit (Zymo Research, Orange, CA, USA). Unmethylated cytosines were converted to uracils while methylated cytosines remained unchanged^73^. Bisulfite-converted DNA samples were then used in the custom-designed Illumina GoldenGate DNA methylation assay.

### Illumina GoldenGate DNA methylation assay

The design of the customized Illumina GoldenGate DNA Methylation array was described in our previous publication^50^. Supplementary Table SI contains information on 384 CpGs in the promoter regions of 82 candidate genes involved in the opioidergic system (8 genes/51 CpGs), the serotonergic system (11 genes/52 CpGs), the dopaminergic system (8 genes/45 CpGs), the GABAergic system (13 genes/42 CpGs), the glutamatergic system (7 genes/45 CpGs), the cannabinoid system (*CNR1*: 3 CpGs), and the cholinergic system (6 genes/34 CpGs), as well as alcohol metabolism (5 genes/14 CpGs), DNA methylation (8 genes/34 CpGs), signal transduction (12 genes/53 CpGs), and several others (*ANKK1*: 4 CpGs; *NCAM1*: 4 CpGs; *TTC12*: 3 CpGs). The hybridization probes were highly specific for these 384 CpGs [GGMAScore (mean ± S.D.): 0.85 ± 0.06].

After bisulfite conversion of genomic DNA, the remaining methylation assay steps were the same as those previously described^50^. Image processing and intensity data extraction were performed using the Illumina GenomeStudio^TM^ Methylation Module v.1.0 Software. The background normalization algorithm was used to minimize background variation within the array by using built-in negative control signals. The methylation level (defined as β) of each individual CpG site was estimated as the ratio of intensities between methylated and unmethylated alleles. The β value was calculated as β = [Max(Cy5,0)]/[Max (Cy3,0) + Max(Cy5,0) + α]. A constant offset α (by default, α = 100) was added to the denominator to adjust β values when both methylated and unmethylated probe intensities were low. The β value ranges from 0 (*i.e.*, completely unmethylated) to 1 (*i.e.*, completely methylated).

### Statistical and bioinformatics analyses

The statistical analysis of DNA methylation data was implemented in R (version 3.1.3) (http://www.r-project.org/). To identify differentially methylated CpGs in subjects with AD and ND codependence, we used the linear mixed-effects model handled by the R package CpGassoc, which was designed for analyzing the association between methylation at CpG sites across the genome and a phenotype of interest^74^. In the linear mixed-effect model built-in CpGassoc, the methylation level of CpGs was the response variable, the status of AD-ND codependence and other covariates (age, sex, and ancestry proportions) were fitted *via* fixed effects, and batch factor (referring to methylation chips) was fitted as a random effect. A *post hoc* power analysis by R package SIMR^75^ served to calculate the power for a linear mixed model based on the observed data structure. For both EAs and AAs, we evaluated the sample power by varying effect sizes and using 100 simulations.

The interactive effect of differentially methylated CpGs and nearby single nucleotide polymorphisms (SNPs) on AD-ND codependence risk was examined using the generalized linear mixed-effects model by the lme4 package in R (http://CRAN.R-project.org/package=lme4). Although the correlation of CpGs and SNPs can be detected when they are within one million bases apart, the strength of correlation between CpGs and SNPs is dramatically decreased when their distance is increased^67^. Here, we just included those SNPs that are 50 kb away from the CpG site in the SNP-CpG interaction analysis. SNP genotype data were generated *via* the Illumina HumanOmni1-Quad v1.0 microarray, as described in our previous GWAS on AD^26^ and ND^37^. Genotype data were cleaned with a commonly used procedure, that is, SNPs were excluded if they met any of following criteria: 1) minor allele frequency (MAF) was <0.05; 2) missing genotyping rate was >10%; or 3) *P* value of Hardy-Weinberg disequilibrium test was <1.0 × 10^−3^. The association of SNP genotypes (in an additive model) and CpG methylation levels was analyzed using PLINK^76^. For both EAs and AAs, linkage disequilibrium (LD) plots of SNPs near CpGs were generated by R package LDheatmap^77^.

Additionally, the function of differentially methylated promoter CpGs was predicted by bioinformatics programs. The online program PROMO^78^,^79^ was applied to predict whether differentially methylated promoter CpGs were located in transcription factor binding sites (TFBS) as defined in the TRANSFAC database^80^. The USCS Genome Browser (http://genome.ucsc.edu) was used to query whether differentially methylated promoter CpGs were located in DNase hypersensitivity sites (DHSs) or in chromosomal regions that were associated with H3K27Ac mark (which are often found near active regulatory elements).

## Additional Information

**How to cite this article:** Xu, H. *et al*. Alcohol and nicotine codependence-associated DNA methylation changes in promoter regions of addiction-related genes. *Sci. Rep.* **7**, 41816; doi: 10.1038/srep41816 (2017).

**Publisher's note:** Springer Nature remains neutral with regard to jurisdictional claims in published maps and institutional affiliations.

## Supplementary Material

Supplementary Information

## Figures and Tables

**Table 1:** Differentially methylated CpGs in subjects with alcohol-nicotine codependence.

CpGs	Genes	β (Mean ± S.D.)	Effect size	*P* values
African Americans (n = 256)				
cg27531267	*HTR2B*	0.043 ± 0.013	−0.005	7.2 × 10^−5^
European Americans (n = 196)				
cg27531267	*HTR2B*	0.038 ± 0.012	0.006	2.1 × 10^−7^
cg02156408	*HTR2C*	0.088 ± 0.023	0.009	8.0 × 10^−6^
cg08989585	*HTR3A*	0.170 ± 0.016	0.007	1.5 × 10^−5^
cg14534584	*SLC6A4*	0.054 ± 0.017	0.007	8.1 × 10^−5^
cg05421426	*DRD2*	0.090 ± 0.022	0.008	4.0 × 10^−6^
cg08079114	*DRD4*	0.054 ± 0.013	0.006	4.5 × 10^−5^
cg00037218	*SLC6A3*	0.033 ± 0.014	0.006	8.2 × 10^−5^
cg21074850	*GABRB1*	0.059 ± 0.019	0.007	1.3 × 10^−4^
cg02095443	*GABRB2*	0.061 ± 0.016	0.006	3.8 × 10^−5^
cg04123893	*GAD1*	0.296 ± 0.032	0.012	9.5 × 10^−5^
cg01706569	*OPRD1*	0.045 ± 0.016	0.007	4.3 × 10^−5^
cg07344165	*OPRK1*	0.034 ± 0.015	0.006	8.9 × 10^−5^
cg26106216	*PENK*	0.028 ± 0.014	0.007	5.7 × 10^−7^
cg12505522	*RGS17*	0.056 ± 0.017	0.007	6.7 × 10^−5^
cg14313206	*NCAM1*	0.049 ± 0.012	0.005	7.7 × 10^−6^
cg21572351	*NCAM1*	0.141 ± 0.019	0.008	8.2 × 10^−6^
cg21372728	*MBD3*	0.051 ± 0.014	0.008	5.6 × 10^−9^

**Table 2:** Interactive effects of *HTR2B* cg27531267 with 13 nearby SNPs on alcohol and nicotine codependence.

SNP	CpG-SNP distance (bp)	Effects of SNPs on AD-ND codependence				cg27531267-SNP interactions on AD-ND codependence			
		Estimate	Std. error	Z	*P*	Estimate	Std. error	Z	*P*
Interactions of cg27531267 and nearby SNPs in African Americans (AAs):									
rs6436999	−49250	−0.396	0.913	−0.433	0.665	−1.053	20.899	−0.050	0.960
rs2303357	−41796	−0.160	0.735	−0.218	0.828	6.543	16.433	0.398	0.691
rs16827801	22590	0.500	0.775	0.645	0.519	−10.499	17.543	−0.598	0.550
rs10194776	−11723	−0.467	0.964	−0.484	0.628	0.907	22.385	0.041	0.968
rs17619588	−19567	0.468	0.841	0.557	0.578	−8.995	19.191	−0.469	0.639
kgp14695769	−18326	−1.186	1.350	−0.878	0.380	25.791	29.761	0.867	0.386
rs16827784	−10965	−1.203	1.352	−0.890	0.374	26.079	29.803	0.875	0.382
rs10199752	−22188	0.552	0.916	0.603	0.547	−1.156	20.849	−0.055	0.956
rs1549339	−8916	−0.297	0.776	−0.383	0.702	9.857	17.497	0.563	0.573
rs10187149	−1371	0.496	0.778	0.637	0.524	−10.489	17.673	−0.594	0.553
rs4635521	29520	−0.539	0.848	−0.635	0.525	4.375	19.022	0.230	0.818
rs13430407	43687	−0.552	0.916	−0.603	0.547	1.156	20.849	0.055	0.956
rs12468767	45101	2.926	1.655	1.768	0.077	−68.597	39.852	−1.721	0.085
Interactions of cg27531267 and nearby SNPs in European Americans (EAs):									
rs6436999	−49250	−5.551	7.974	−0.696	0.486	177.230	238.254	0.744	0.457
rs2303357	−41796	−4.473	7.842	−0.570	0.568	143.319	232.680	0.616	0.538
rs16827801	22590	5.283	6.727	0.785	0.430	−173.067	200.339	−0.864	0.380
rs10194776	−11723	−2.252	8.112	−0.278	0.781	85.800	241.817	0.355	0.722
rs17619588	−19567	1.276	9.212	0.138	0.890	−89.607	270.476	−0.331	0.740
kgp14695769	−18326	N/A	N/A	N/A	N/A	N/A	N/A	N/A	N/A
rs16827784	−10965	N/A	N/A	N/A	N/A	N/A	N/A	N/A	N/A
rs10199752	−22188	4.366	7.833	0.557	0.577	−140.073	232.332	−0.603	0.547
rs1549339	−8916	−4.472	7.838	−0.571	0.568	143.368	232.103	0.618	0.537
rs10187149	−1371	5.182	7.409	0.699	0.484	−169.876	221.410	−0.767	0.443
rs4635521	29520	−5.112	7.429	−0.688	0.491	167.447	222.060	0.754	0.451
rs13430407	43687	−4.366	7.833	−0.557	0.577	140.073	232.333	0.603	0.547
rs12468767	45101	−0.250	9.311	−0.027	0.979	−12.922	279.219	−0.046	0.963

**Table 3:** Function prediction of alcohol-nicotine codependence-associated CpGs.

CpGs	Genes	Chr.	Coordinate	DNA Sequences around CpGs	aTF core binding sites	bDHSs	cH3K27Ac
cg01706569	OPRD1	1	29011768	GTGGGGATCA[**C**G]AACTTGAGAC		Yes	No
cg04123893	GAD1	2	171380892	AACCTTCAAA[**C**G]TGATTAATCA		Yes	Yes
cg27531267	HTR2B	2	231699986	CACACATACA[**C**G]CACACACACG		Yes	Yes
cg21074850	GABRB1	4	46729246	ACATGGTCTC[**C**G]AAGTGAATAT	TFII-I (GTCTC**C**)	No	No
cg00037218	SLC6A3	5	1498390	CCCCGGCCCC[**C**G]CCCCTGCGCC	E2F-1 (GCCCC**C**GC);	No	No
					Sp1 (CCCC**C**GCCCC)		
cg02095443	GABRB2	5	160908531	TGGCGGCAGG[**C**G]GCGGAAGTAG	AP-2alphaA (GCAGG**C**);	Yes	No
					Elk-1 (**C**GGCGGAAG)		
cg12505522	RGS17	6	153494537	CTGCCTGCTC[**C**G]GGTCCCGGAG		Yes	Yes
cg07344165	OPRK1	8	54326330	ACAGGGAGAA[**C**G]GACTTCTCGC	FOXP3 (GAGAA**C**)	Yes	No
cg26106216	PENK	8	57521167	AGGGGATCGT[**C**G]AGCAAAAGCC	NF-kappaB1 (GGGGATCGT**C**G)	Yes	No
cg08079114	DRD4	11	627981	CCCTGGCTGC[**C**G]TGGGACACAC	ENKTF-1 (TGGCTGC**C**)	Yes	No
cg21572351	NCAM1	11	112335975	GGGCAGATCA[**C**G]AGGTCAGGAG	GR-alpha (**C**GAGGTCAGG);	No	Yes
					COUP-TF1 (CA**C**GAGGTCAGGA)		
cg14313206	NCAM1	11	112337308	CCCGGGCCAG[**C**G]CAAGGATCTC	GCF (GGCCAG**C**GC)	Yes	Yes
cg05421426	DRD2	11	112850463	ACCGTGGGAG[**C**G]GGAGAATCCA	E2F-1 (G**C**GGGAGA)	Yes	No
cg08989585	HTR3A	11	113350239	GCAGTGATTG[**C**G]CCACTGCACT	C/EBPbeta (TTG**C**);	Yes	No
					C/EBPalpha (GATTG**C**G);		
					ENKTF-1 (TTG**C**GCCA)		
cg14534584	SLC6A4	17	25587232	CCTGCCGCCC[**C**G]CGCCCACAGG	Pax-5 (GCCGCCC**C**GCGCCC);	Yes	No
					p53 (GCCGCCC**C**GCGCCC);		
					Sp1 (CTGCCGCCC**C**);		
					GCF (GCCC**C**GCGC);		
					ETF (GCCC**C**GCGCCC)		
cg21372728	MBD3	19	1543111	CCCTGCTCCC[**C**G]AAATCCCGGC		Yes	Yes
cg02156408	HTR2C	X	113725033	GGCCTTCGTC[**C**G]TTTAGAGTAG		Yes	No

^a^Transcription factor (TF) binding sites predicted by PROMO.

^b^DNase I Hypersensitivity sites (DHSs) from ENCODE (95 cell types) by the UCSC Genome Browser.

^c^H3K27Ac mark (often found near active regulatory elements) on 7 cell lines from ENCODE by the UCSC Genome Browser.
