# Supplementary material for: Alcohol and nicotine codependence-associated DNA methylation changes in promoter regions of addiction-related genes
Source: Sci Rep. 2017 Feb 6;7:41816. doi: 10.1038/srep41816 (PMC5292964; doi:10.1038/srep41816)
Supplement: Supplementary Information [file srep41816-s1.pdf]

Alcohol and nicotine codependence-associated DNA methylation changes

in promoter regions of addiction-related genes

(Supplementary information)

Hongqin Xu, Fan Wang, Henry R. Kranzler, Joel Gelernter, Huiping Zhang

Table S1. Association of 384 promoter CpGs (in 82 genes) and alcohol-nicotine codependence in African Americans (AAs)

Table S2. Association of 384 Promoter CpGs (in 82 Genes) and alcohol-nicotine codependence in European Americans (EAs)

Table S3. Information of 384 CpGs in 82 candidate genes

Figure S1. Linkage disequilibrium (LD) plots of 13 SNPs near CpG cg27531267 in gene *HTR2B*

Table S1. Association of 384 promoter CpGs (in 82 genes) and alcohol-nicotine codependence in African Americans (AAs)
(CpGs in red had nominally significant associations with AD-ND codependence in both AAs and EAs)

CpGs	Chr.	Genes	Mean β (Cases)	S.D. (Cases)	Mean β (Controls)	S.D. (Controls)	T statistic	*P* value	Effect size
cg27531267	2	HTR2B	0.041	0.011	0.045	0.015	-4.041	0.0001	-0.005
cg07316621	11	NCAM1	0.011	0.006	0.013	0.008	-3.756	0.0002	-0.003
cg07975378	17	PPP1R1B	0.044	0.013	0.049	0.015	-3.721	0.0002	-0.005
cg17466970	2	GAD1	0.037	0.014	0.042	0.015	-3.694	0.0003	-0.005
cg06281629	19	MBD3	0.009	0.003	0.010	0.005	-3.688	0.0003	-0.002
cg10390074	18	MBD1	0.009	0.002	0.010	0.002	-3.684	0.0003	-0.001
cg20787301	19	GRIN2D	0.433	0.090	0.471	0.090	-3.646	0.0003	-0.038
cg11530112	6	CNR1	0.706	0.114	0.659	0.117	3.527	0.0005	0.048
cg23209660	17	PPP1R9B	0.028	0.011	0.031	0.013	-3.419	0.0007	-0.004
cg24774208	10	PPA1	0.015	0.009	0.018	0.011	-3.400	0.0008	-0.003
cg01702186	19	GRIN2D	0.023	0.010	0.026	0.011	-3.388	0.0008	-0.003
cg09200586	19	GRIN2D	0.041	0.012	0.044	0.012	-3.364	0.0009	-0.004
cg25997474	4	ADH1C	0.076	0.018	0.083	0.021	-3.297	0.0011	-0.008
cg25204262	11	DRD4	0.783	0.054	0.769	0.063	3.293	0.0011	0.020
cg26524348	17	PPP1R9B	0.024	0.011	0.027	0.012	-3.274	0.0012	-0.004
cg01706569	1	OPRD1	0.045	0.014	0.050	0.017	-3.258	0.0013	-0.005
cg08642921	19	DNMT1	0.019	0.010	0.021	0.012	-3.231	0.0014	-0.003
cg06365654	20	CHRNA4	0.020	0.009	0.022	0.011	-3.218	0.0015	-0.003
cg02156408	23	HTR2C	0.092	0.023	0.104	0.025	-3.205	0.0015	-0.008
cg00243951	11	DRD2	0.012	0.004	0.013	0.006	-3.195	0.0016	-0.002
cg26106216	8	PENK	0.031	0.013	0.035	0.018	-3.121	0.0020	-0.005
cg03461962	6	RGS17	0.014	0.005	0.015	0.007	-3.119	0.0020	-0.002
cg11829658	20	CHRNA4	0.470	0.103	0.502	0.096	-3.103	0.0021	-0.031
cg05893218	12	GRIN2B	0.047	0.013	0.049	0.014	-3.023	0.0028	-0.004
cg22646454	5	HTR1A	0.030	0.011	0.033	0.014	-3.014	0.0029	-0.004
cg20574282	3	MBD4	0.029	0.008	0.032	0.008	-2.992	0.0031	-0.003
cg20518314	2	DNMT3A	0.016	0.010	0.018	0.012	-2.982	0.0032	-0.003
cg13890276	5	HTR1A	0.047	0.017	0.050	0.016	-2.968	0.0033	-0.005

cg03446957	22	COMT	0.005	0.006	0.007	0.007	-2.916	0.0039	-0.002
cg17111401	11	DRD4	0.437	0.093	0.423	0.086	2.901	0.0041	0.030
cg11861961	5	SLC6A3	0.027	0.012	0.030	0.014	-2.836	0.0050	-0.004
cg12570007	4	ADH5	0.028	0.012	0.032	0.014	-2.810	0.0054	-0.004
cg13887561	6	OPRM1	0.030	0.011	0.033	0.013	-2.787	0.0058	-0.003
cg25483501	15	CHRNA5	0.074	0.019	0.080	0.020	-2.761	0.0062	-0.006
cg02585344	16	GRIN2A	0.039	0.012	0.042	0.014	-2.758	0.0063	-0.004
cg22872776	20	OPRL1	0.022	0.012	0.025	0.014	-2.757	0.0063	-0.003
cg12505522	6	RGS17	0.059	0.017	0.062	0.017	-2.754	0.0064	-0.005
cg26237037	6	RGS17	0.025	0.009	0.028	0.010	-2.747	0.0065	-0.003
cg21074850	4	GABRB1	0.062	0.017	0.066	0.021	-2.743	0.0066	-0.005
cg11153544	7	DDC	0.069	0.030	0.089	0.040	-2.733	0.0068	-0.012
cg02651732	5	SLC6A3	0.031	0.010	0.034	0.012	-2.717	0.0071	-0.003
cg09864658	9	GRIN1	0.083	0.021	0.087	0.022	-2.702	0.0074	-0.006
cg06671711	8	PENK	0.015	0.010	0.017	0.012	-2.682	0.0078	-0.003
cg17987474	15	CHRNA3	0.033	0.019	0.038	0.022	-2.676	0.0080	-0.005
cg05884848	17	GRIN2C	0.019	0.013	0.022	0.014	-2.654	0.0085	-0.003
cg07344165	8	OPRK1	0.036	0.014	0.041	0.019	-2.650	0.0086	-0.004
cg08079114	11	DRD4	0.055	0.014	0.060	0.015	-2.600	0.0099	-0.004
cg22848316	15	CHRNA3	0.025	0.013	0.028	0.015	-2.597	0.0100	-0.004
cg00037218	5	SLC6A3	0.035	0.013	0.038	0.016	-2.593	0.0101	-0.004
cg22089561	2	GAD1	0.058	0.012	0.061	0.014	-2.593	0.0101	-0.004
cg26187884	10	GAD2	0.012	0.007	0.014	0.010	-2.592	0.0102	-0.002
cg00848742	2	CREB1	0.769	0.059	0.782	0.063	-2.589	0.0102	-0.016
cg00001938	17	GRIN2C	0.028	0.016	0.031	0.018	-2.563	0.0110	-0.004
cg17108064	15	CHRNA5	0.079	0.031	0.067	0.025	2.496	0.0132	0.009
cg03896970	20	OPRL1	0.034	0.011	0.036	0.013	-2.476	0.0140	-0.003
cg17771682	15	GABRB3	0.016	0.011	0.018	0.013	-2.472	0.0141	-0.003
cg20650766	15	CHRNA3	0.038	0.012	0.041	0.015	-2.472	0.0141	-0.003
cg19018954	19	DNMT1	0.020	0.013	0.023	0.015	-2.466	0.0144	-0.003
cg17137171	11	TTC12	0.817	0.040	0.832	0.037	-2.440	0.0154	-0.012
cg15244006	20	OPRL1	0.059	0.014	0.063	0.015	-2.412	0.0166	-0.004

cg22358797	18	MBD2	0.056	0.012	0.058	0.013	-2.406	0.0169	-0.003
cg14544856	2	CREB1	0.038	0.013	0.041	0.012	-2.386	0.0178	-0.003
cg05876300	23	MAOB	0.024	0.013	0.031	0.014	-2.331	0.0206	-0.003
cg04055210	23	GABRA3	0.026	0.011	0.032	0.014	-2.323	0.0210	-0.003
cg19713819	10	GAD2	0.075	0.023	0.078	0.023	-2.318	0.0213	-0.006
cg00582628	8	RGS20	0.455	0.060	0.482	0.058	-2.318	0.0213	-0.016
cg12966714	19	MBD3	0.029	0.013	0.032	0.015	-2.307	0.0219	-0.003
cg11521282	15	CHRNA5	0.015	0.004	0.016	0.006	-2.303	0.0221	-0.001
cg08989585	11	HTR3A	0.165	0.017	0.172	0.020	-2.290	0.0229	-0.004
cg11500467	10	GAD2	0.017	0.007	0.018	0.009	-2.285	0.0232	-0.002
cg02928015	9	DBH	0.339	0.060	0.329	0.059	2.269	0.0242	0.012
cg18574886	3	MBD4	0.013	0.008	0.014	0.009	-2.255	0.0251	-0.002
cg24591506	16	GRIN2A	0.027	0.010	0.029	0.013	-2.248	0.0255	-0.003
cg12215457	6	HTR1B	0.097	0.021	0.090	0.020	2.244	0.0258	0.005
cg06777434	11	NCAM1	0.011	0.007	0.011	0.008	-2.232	0.0265	-0.002
cg04941592	23	MECP2	0.308	0.034	0.307	0.036	2.215	0.0277	0.008
cg19491443	23	HTR2C	0.520	0.093	0.557	0.069	-2.208	0.0282	-0.022
cg17820491	6	HTR1B	0.038	0.017	0.034	0.014	2.170	0.0310	0.005
cg13752831	10	PPA1	0.022	0.006	0.024	0.008	-2.153	0.0323	-0.002
cg13941250	12	ALDH2	0.941	0.011	0.939	0.017	2.144	0.0331	0.004
cg06319384	15	CHRNB4	0.077	0.018	0.081	0.016	-2.140	0.0334	-0.004
cg12040841	8	PNOC	0.606	0.124	0.628	0.123	-2.129	0.0343	-0.024
cg05421426	11	DRD2	0.084	0.018	0.089	0.020	-2.125	0.0346	-0.004
cg20550533	23	MAOB	0.069	0.066	0.099	0.053	2.110	0.0359	0.009
cg02395672	6	CNR1	0.444	0.038	0.426	0.042	2.108	0.0361	0.010
cg19391527	8	PNOC	0.699	0.050	0.715	0.058	-2.098	0.0370	-0.014
cg07283003	6	HTR1B	0.793	0.026	0.784	0.040	2.092	0.0375	0.009
cg12296643	6	FYN	0.041	0.013	0.043	0.013	-2.091	0.0376	-0.003
cg02567788	10	PPA1	0.025	0.008	0.027	0.009	-2.076	0.0390	-0.002
cg17388934	18	MBD2	0.010	0.007	0.011	0.008	-2.069	0.0396	-0.002
cg03476087	10	GAD2	0.022	0.013	0.024	0.015	-2.042	0.0423	-0.003
cg16024485	20	OPRL1	0.031	0.011	0.034	0.012	-2.028	0.0437	-0.002

cg00112517	17	PPP1R1B	0.027	0.011	0.029	0.014	-2.024	0.0441	-0.003
cg27321505	8	PENK	0.033	0.011	0.034	0.014	-2.014	0.0452	-0.003
cg19289837	2	GAD1	0.027	0.014	0.030	0.015	-2.006	0.0461	-0.003
cg23869328	6	RGS17	0.010	0.004	0.011	0.005	-2.004	0.0462	-0.001
cg06063362	1	OPRD1	0.323	0.038	0.313	0.039	2.003	0.0463	0.009
cg04550775	12	GRIN2B	0.010	0.004	0.010	0.005	-2.003	0.0463	-0.001
cg01668758	2	CREB1	0.126	0.027	0.134	0.024	-1.984	0.0484	-0.005
cg26258452	17	SLC6A4	0.198	0.028	0.196	0.030	1.978	0.0491	0.006
cg15490013	5	DRD1	0.036	0.011	0.038	0.011	-1.977	0.0492	-0.003
cg11880892	10	PPA1	0.014	0.004	0.015	0.003	-1.974	0.0496	-0.001
cg10615371	15	CHRNA5	0.174	0.040	0.161	0.041	1.972	0.0498	0.010
cg00295802	5	SLC6A3	0.463	0.043	0.454	0.053	1.964	0.0508	0.009
cg01884662	15	CHRNA7	0.108	0.025	0.113	0.023	-1.954	0.0519	-0.005
cg01045241	2	DNMT3A	0.268	0.031	0.276	0.028	-1.951	0.0522	-0.007
cg08684483	2	GAD1	0.013	0.009	0.015	0.009	-1.951	0.0523	-0.002
cg23463131	16	GRIN2A	0.033	0.009	0.035	0.010	-1.947	0.0528	-0.002
cg17354190	17	GRIN2C	0.014	0.010	0.015	0.011	-1.920	0.0560	-0.002
cg24285775	4	ADH5	0.086	0.019	0.090	0.018	-1.916	0.0566	-0.004
cg06031989	6	HTR1B	0.056	0.018	0.050	0.016	1.915	0.0567	0.004
cg03109047	9	GRIN1	0.017	0.008	0.018	0.008	-1.908	0.0576	-0.002
cg26647484	2	DNMT3A	0.019	0.008	0.020	0.009	-1.888	0.0602	-0.002
cg22515311	4	DRD5	0.273	0.028	0.262	0.035	1.885	0.0606	0.007
cg24972720	7	CHRM2	0.033	0.014	0.035	0.016	-1.877	0.0617	-0.003
cg14534584	17	SLC6A4	0.055	0.015	0.058	0.018	-1.872	0.0624	-0.003
cg09488889	20	PDYN	0.870	0.078	0.848	0.083	1.831	0.0684	0.019
cg19518651	5	GABRA1	0.009	0.003	0.009	0.004	-1.822	0.0698	-0.001
cg21572351	11	NCAM1	0.143	0.021	0.146	0.022	-1.821	0.0699	-0.004
cg02340737	4	GABRA2	0.019	0.013	0.021	0.014	-1.820	0.0701	-0.003
cg17659879	22	MAPK1	0.018	0.008	0.019	0.008	-1.814	0.0710	-0.001
cg15268261	13	HTR2A	0.411	0.031	0.404	0.041	1.813	0.0711	0.007
cg18219951	5	GABRG2	0.032	0.011	0.033	0.014	-1.802	0.0728	-0.002
cg14905768	1	OPRD1	0.018	0.007	0.020	0.008	-1.802	0.0729	-0.002

cg17128529	20	PDYN	0.421	0.060	0.434	0.058	-1.786	0.0754	-0.013
cg08754521	8	PENK	0.029	0.024	0.020	0.012	1.771	0.0778	0.004
cg04312520	17	GRIN2C	0.217	0.024	0.213	0.028	1.758	0.0800	0.004
cg26300748	5	HTR1A	0.032	0.013	0.033	0.015	-1.741	0.0831	-0.003
cg10466664	5	GABRG2	0.029	0.013	0.026	0.011	1.738	0.0835	0.003
cg04000318	8	PNOC	0.373	0.131	0.407	0.151	-1.736	0.0838	-0.033
cg12235279	4	GABRG1	0.704	0.062	0.714	0.064	-1.729	0.0851	-0.015
cg00618092	2	HTR2B	0.576	0.054	0.560	0.070	1.724	0.0860	0.014
cg19135956	15	CHRNB4	0.753	0.032	0.749	0.050	1.715	0.0876	0.009
cg16769226	5	DRD1	0.406	0.041	0.400	0.039	1.713	0.0881	0.009
cg20648561	6	HTR1B	0.129	0.045	0.138	0.051	-1.704	0.0897	-0.010
cg09897477	20	CHRNA4	0.024	0.015	0.025	0.018	-1.693	0.0918	-0.004
cg20467969	4	GABRA2	0.079	0.018	0.081	0.017	-1.690	0.0924	-0.003
cg05989757	22	MAPK1	0.004	0.004	0.006	0.005	-1.686	0.0932	-0.001
cg01579505	17	PPP1R9B	0.037	0.015	0.038	0.015	-1.673	0.0956	-0.003
cg14738521	19	DNMT1	0.012	0.005	0.011	0.004	1.673	0.0957	0.001
cg02577095	2	POMC	0.018	0.010	0.020	0.012	-1.646	0.1012	-0.002
cg03088662	16	GRIN2A	0.013	0.013	0.010	0.011	1.622	0.1062	0.003
cg16941825	4	DRD5	0.035	0.013	0.037	0.016	-1.620	0.1066	-0.002
cg12511279	18	MBD2	0.807	0.031	0.802	0.043	1.619	0.1067	0.008
cg10400239	20	PDYN	0.340	0.069	0.330	0.057	1.617	0.1073	0.010
cg15130599	5	DRD1	0.229	0.033	0.222	0.033	1.610	0.1088	0.006
cg08876474	15	CHRNA5	0.009	0.003	0.009	0.003	-1.608	0.1092	-0.001
cg05784269	8	RGS20	0.270	0.033	0.278	0.029	-1.606	0.1096	-0.006
cg24462132	11	TTC12	0.039	0.013	0.041	0.013	-1.605	0.1099	-0.002
cg16865650	6	OPRM1	0.406	0.100	0.416	0.115	-1.600	0.1109	-0.018
cg00331892	6	FYN	0.031	0.014	0.033	0.015	-1.588	0.1136	-0.003
cg23155627	11	PTPN5	0.052	0.012	0.053	0.013	-1.587	0.1140	-0.002
cg26041285	17	GRIN2C	0.053	0.027	0.049	0.026	1.568	0.1181	0.005
cg12139952	4	GABRG1	0.390	0.098	0.396	0.106	-1.560	0.1201	-0.022
cg05293338	5	HTR1A	0.255	0.026	0.263	0.030	-1.559	0.1205	-0.005
cg18584905	17	SLC6A4	0.135	0.042	0.160	0.039	-1.557	0.1208	-0.005

cg23404860	11	DRD4	0.214	0.046	0.209	0.046	1.554	0.1216	0.008
cg22921295	5	DRD1	0.040	0.011	0.042	0.010	-1.539	0.1251	-0.002
cg05931924	4	DRD5	0.031	0.020	0.033	0.019	-1.515	0.1312	-0.003
cg25195998	11	DRD2	0.046	0.013	0.048	0.014	-1.503	0.1341	-0.003
cg15291052	15	CHRNA7	0.035	0.012	0.037	0.013	-1.502	0.1346	-0.002
cg02945241	19	GRIN2D	0.059	0.015	0.061	0.015	-1.494	0.1366	-0.002
cg14109444	3	MBD4	0.060	0.014	0.064	0.015	-1.487	0.1383	-0.002
cg14009433	23	MAOA	0.077	0.044	0.112	0.037	-1.487	0.1384	-0.003
cg11227734	2	POMC	0.053	0.018	0.054	0.015	-1.480	0.1401	-0.003
cg20346122	3	MBD4	0.105	0.019	0.107	0.017	-1.473	0.1420	-0.003
cg15587034	6	HTR1B	0.011	0.004	0.011	0.006	-1.473	0.1422	-0.001
cg22851911	3	MBD4	0.096	0.021	0.100	0.021	-1.437	0.1521	-0.003
cg01163502	1	OPRD1	0.690	0.046	0.681	0.068	1.428	0.1546	0.012
cg24799561	5	GABRA1	0.017	0.005	0.018	0.006	-1.407	0.1607	-0.001
cg25304536	6	HTR1B	0.057	0.016	0.058	0.016	-1.396	0.1642	-0.002
cg06711394	5	GABRB2	0.059	0.015	0.060	0.017	-1.395	0.1644	-0.003
cg11722044	23	MECP2	0.434	0.086	0.489	0.064	-1.394	0.1645	-0.011
cg05788582	18	MBD1	0.675	0.063	0.668	0.050	1.386	0.1672	0.010
cg22498099	15	CHRNA5	0.006	0.004	0.007	0.005	-1.379	0.1691	-0.001
cg12216825	6	HTR1B	0.016	0.006	0.016	0.009	-1.369	0.1724	-0.001
cg05286097	17	SLC6A4	0.194	0.030	0.196	0.028	1.347	0.1793	0.004
cg22917359	22	MAPK1	0.865	0.040	0.873	0.041	-1.337	0.1824	-0.007
cg05964444	17	SLC6A4	0.068	0.019	0.069	0.020	-1.328	0.1856	-0.003
cg01768936	11	PTPN5	0.209	0.025	0.215	0.024	-1.327	0.1859	-0.004
cg09356486	23	MAOA	0.183	0.027	0.187	0.026	-1.319	0.1884	-0.004
cg01709006	5	GABRG2	0.067	0.018	0.065	0.020	-1.304	0.1935	-0.003
cg17636534	23	HTR2C	0.071	0.047	0.094	0.042	1.296	0.1962	0.005
cg03490309	20	CHRNA4	0.095	0.024	0.099	0.024	-1.285	0.2002	-0.003
cg20203806	11	DRD2	0.126	0.023	0.123	0.023	1.283	0.2008	0.003
cg23079189	11	PTPN5	0.583	0.080	0.575	0.088	1.277	0.2027	0.009
cg15606313	18	MBD1	0.012	0.006	0.013	0.006	-1.273	0.2041	-0.001
cg01677874	5	GABRB2	0.105	0.017	0.107	0.018	-1.273	0.2042	-0.002

cg10794519	17	GRIN2C	0.096	0.019	0.098	0.021	-1.272	0.2047	-0.003
cg22531992	19	MBD3	0.700	0.062	0.696	0.061	1.262	0.2082	0.009
cg00779299	5	DRD1	0.864	0.039	0.872	0.037	-1.262	0.2082	-0.006
cg15232722	8	OPRK1	0.445	0.067	0.429	0.072	1.261	0.2087	0.012
cg06841599	23	HTR2C	0.060	0.029	0.070	0.028	1.260	0.2090	0.004
cg20621129	11	HTR3A	0.308	0.051	0.302	0.049	1.253	0.2114	0.007
cg21372728	19	MBD3	0.052	0.014	0.054	0.016	-1.250	0.2125	-0.002
cg08339494	19	DNMT1	0.013	0.022	0.010	0.016	1.246	0.2138	0.003
cg13245417	11	PTPN5	0.009	0.004	0.009	0.005	-1.235	0.2181	-0.001
cg06940827	9	DBH	0.529	0.050	0.544	0.059	-1.232	0.2191	-0.008
cg20133817	9	GRIN1	0.077	0.021	0.073	0.021	1.223	0.2225	0.003
cg17915420	2	POMC	0.295	0.021	0.293	0.029	1.216	0.2253	0.003
cg08792950	7	DDC	0.898	0.030	0.896	0.040	1.205	0.2293	0.006
cg26566103	17	RGS9	0.578	0.049	0.572	0.053	1.205	0.2295	0.008
cg10670893	17	PPP1R1B	0.219	0.074	0.234	0.065	-1.202	0.2304	-0.009
cg01928350	6	RGS17	0.122	0.026	0.122	0.029	-1.199	0.2317	-0.004
cg14313206	11	NCAM1	0.050	0.014	0.050	0.012	-1.183	0.2379	-0.002
cg17259718	9	GRIN1	0.202	0.052	0.197	0.049	-1.178	0.2400	-0.008
cg17929169	6	FYN	0.008	0.004	0.009	0.004	-1.170	0.2431	-0.001
cg12065362	11	DRD4	0.199	0.049	0.194	0.050	1.168	0.2439	0.007
cg15234319	15	GABRB3	0.287	0.065	0.273	0.065	1.166	0.2447	0.009
cg22196019	22	COMT	0.009	0.005	0.010	0.006	-1.163	0.2458	-0.001
cg11722562	20	OPRL1	0.118	0.059	0.107	0.030	1.149	0.2516	0.007
cg19126369	2	GAD1	0.031	0.013	0.032	0.015	-1.136	0.2569	-0.001
cg24723845	6	FYN	0.074	0.012	0.075	0.012	-1.135	0.2577	-0.002
cg20494803	4	DRD5	0.421	0.089	0.392	0.092	1.118	0.2649	0.014
cg16164153	20	DNMT3B	0.010	0.006	0.010	0.007	-1.105	0.2703	-0.001
cg01732618	4	GABRA4	0.029	0.008	0.029	0.007	-1.102	0.2717	-0.001
cg08289627	18	MBD1	0.568	0.053	0.561	0.061	1.100	0.2724	0.007
cg23137284	15	CHRNA5	0.090	0.022	0.092	0.019	-1.094	0.2752	-0.003
cg27580859	23	MAOA	0.386	0.053	0.402	0.041	-1.031	0.3037	-0.006
cg16206611	9	ALDH1A1	0.075	0.021	0.072	0.017	1.000	0.3184	0.003

cg07170180	20	DNMT3B	0.042	0.015	0.041	0.013	-0.998	0.3194	-0.002
cg23193606	4	GABRA4	0.012	0.004	0.012	0.006	-0.991	0.3228	-0.001
cg21590372	12	GRIN2B	0.036	0.008	0.037	0.010	-0.989	0.3238	-0.001
cg09883163	23	MAOA	0.078	0.078	0.132	0.069	0.987	0.3247	0.003
cg05807600	4	GABRA2	0.107	0.025	0.109	0.023	-0.978	0.3290	-0.002
cg19590658	11	ANKK1	0.075	0.019	0.075	0.017	-0.974	0.3311	-0.002
cg00314411	20	OPRL1	0.159	0.050	0.151	0.041	0.972	0.3323	0.006
cg23691961	4	GABRG1	0.048	0.024	0.049	0.029	-0.971	0.3328	-0.004
cg16755630	4	GABRB1	0.212	0.029	0.208	0.030	0.969	0.3337	0.003
cg19285359	5	CART	0.033	0.016	0.033	0.011	-0.964	0.3362	-0.002
cg11487886	2	POMC	0.420	0.042	0.419	0.042	0.959	0.3384	0.005
cg25624924	7	CHRM2	0.025	0.008	0.024	0.009	-0.955	0.3404	-0.001
cg20573040	3	SLC6A1	0.025	0.008	0.025	0.008	-0.945	0.3456	-0.001
cg00293936	2	POMC	0.128	0.032	0.130	0.025	-0.938	0.3490	-0.003
cg12902246	20	RGS19	0.196	0.037	0.195	0.033	0.933	0.3518	0.004
cg27643501	12	GRIN2B	0.065	0.015	0.065	0.014	-0.930	0.3536	-0.001
cg06020661	13	HTR2A	0.676	0.086	0.660	0.087	0.928	0.3545	0.011
cg17672157	2	CREB1	0.748	0.096	0.767	0.067	-0.919	0.3588	-0.009
cg04527961	15	CHRNB4	0.031	0.018	0.028	0.018	0.915	0.3613	0.002
cg01378239	10	PPA1	0.494	0.080	0.485	0.071	0.907	0.3655	0.008
cg26927763	5	CART	0.349	0.066	0.343	0.057	0.906	0.3658	0.007
cg19130396	7	CHRM2	0.069	0.018	0.071	0.017	-0.904	0.3672	-0.002
cg16740031	5	HTR1A	0.257	0.032	0.259	0.031	0.902	0.3677	0.003
cg23617770	20	PDYN	0.502	0.076	0.503	0.070	0.899	0.3694	0.009
cg07081615	5	SLC6A3	0.076	0.019	0.078	0.018	-0.886	0.3764	-0.002
cg08411435	17	PPP1R1B	0.589	0.074	0.616	0.069	-0.886	0.3766	-0.008
cg01006616	9	DBH	0.695	0.045	0.691	0.053	0.882	0.3784	0.005
cg14334548	11	PTPN5	0.109	0.027	0.103	0.027	0.873	0.3836	0.003
cg13833700	11	PTPN5	0.011	0.006	0.011	0.006	-0.869	0.3860	-0.001
cg23424273	6	HTR1B	0.009	0.006	0.008	0.006	0.857	0.3924	0.001
cg21272636	11	TPH1	0.863	0.039	0.870	0.044	-0.856	0.3930	-0.004
cg02968741	4	GABRB1	0.009	0.014	0.007	0.008	0.852	0.3953	0.001

cg03593419	4	GABRA4	0.132	0.044	0.136	0.045	-0.842	0.4006	-0.005
cg10000484	5	GABRG2	0.095	0.028	0.094	0.027	0.836	0.4041	0.002
cg05553976	4	DRD5	0.241	0.029	0.238	0.031	0.835	0.4047	0.002
cg01690182	15	GABRB3	0.141	0.019	0.139	0.022	0.820	0.4132	0.002
cg14602957	2	DNMT3A	0.636	0.053	0.626	0.053	0.819	0.4136	0.005
cg17494568	4	DRD5	0.200	0.040	0.195	0.034	0.810	0.4190	0.004
cg02811260	6	HTR1B	0.239	0.050	0.229	0.055	0.808	0.4197	0.006
cg05408953	23	MECP2	0.806	0.049	0.829	0.039	-0.799	0.4252	-0.003
cg02404574	12	GRIN2B	0.013	0.005	0.013	0.004	0.794	0.4281	0.000
cg10592946	15	CHRNA7	0.088	0.022	0.090	0.023	-0.790	0.4306	-0.002
cg23296469	3	SLC6A1	0.033	0.038	0.040	0.037	-0.789	0.4310	-0.004
cg01196743	22	MAPK1	0.008	0.002	0.008	0.002	0.781	0.4355	0.000
cg06315187	2	POMC	0.408	0.032	0.407	0.040	0.779	0.4368	0.003
cg06652524	19	GRIN2D	0.150	0.032	0.155	0.029	-0.776	0.4387	-0.003
cg15123533	17	GRIN2C	0.256	0.036	0.253	0.034	0.773	0.4401	0.003
cg23593402	20	CHRNA4	0.560	0.058	0.560	0.053	0.759	0.4487	0.005
cg03061563	2	HTR2B	0.857	0.043	0.858	0.043	-0.757	0.4501	-0.004
cg15244476	17	PPP1R9B	0.111	0.023	0.113	0.023	-0.756	0.4504	-0.002
cg20391608	22	COMT	0.181	0.045	0.186	0.048	-0.747	0.4559	-0.004
cg20058896	18	MBD1	0.049	0.011	0.050	0.014	-0.747	0.4561	-0.001
cg02225257	15	GABRA5	0.511	0.063	0.522	0.060	-0.736	0.4623	-0.006
cg23798509	3	SLC6A1	0.006	0.003	0.005	0.002	0.735	0.4631	0.000
cg07385443	11	DRD4	0.023	0.007	0.022	0.007	0.729	0.4669	0.001
cg05227791	23	MECP2	0.344	0.027	0.346	0.034	-0.719	0.4731	-0.002
cg22719623	6	OPRM1	0.089	0.021	0.086	0.019	-0.709	0.4788	-0.002
cg01325191	15	CHRNB4	0.893	0.023	0.891	0.029	0.706	0.4808	0.002
cg23759848	5	HTR1A	0.111	0.027	0.112	0.030	-0.702	0.4834	-0.002
cg18655110	12	ALDH2	0.240	0.037	0.244	0.034	-0.690	0.4906	-0.003
cg01115034	5	SLC6A3	0.173	0.036	0.165	0.038	0.685	0.4941	0.004
cg00164724	20	PDYN	0.515	0.055	0.510	0.055	0.683	0.4953	0.005
cg21857413	5	HTR1A	0.043	0.019	0.041	0.016	0.679	0.4980	0.002
cg20038036	11	DRD4	0.588	0.077	0.558	0.083	0.663	0.5082	0.006

cg10236526	8	OPRK1	0.031	0.026	0.032	0.026	-0.660	0.5099	-0.002
cg08354950	5	CART	0.022	0.009	0.023	0.011	-0.658	0.5114	-0.001
cg17610361	4	ADH5	0.082	0.022	0.084	0.023	-0.653	0.5147	-0.001
cg24863581	18	MBD1	0.896	0.026	0.898	0.034	-0.652	0.5153	-0.002
cg12338263	23	MAOB	0.665	0.072	0.645	0.075	-0.641	0.5224	-0.006
cg16405454	11	ANKK1	0.788	0.065	0.797	0.064	-0.633	0.5271	-0.005
cg15408490	12	ALDH2	0.117	0.065	0.129	0.080	-0.632	0.5283	-0.006
cg21239013	12	ALDH2	0.926	0.037	0.930	0.021	-0.601	0.5486	-0.002
cg10944175	9	DBH	0.089	0.021	0.090	0.019	-0.597	0.5514	-0.001
cg18620600	15	CHRNB4	0.750	0.037	0.745	0.047	0.593	0.5536	0.003
cg17484926	11	PTPN5	0.143	0.021	0.142	0.021	0.580	0.5623	0.001
cg04123893	2	GAD1	0.298	0.044	0.298	0.037	0.566	0.5720	0.002
cg24164433	9	GRIN1	0.456	0.047	0.464	0.056	-0.565	0.5723	-0.003
cg06938800	15	CHRNA5	0.109	0.036	0.107	0.036	0.562	0.5747	0.002
cg01840162	8	OPRK1	0.078	0.020	0.081	0.020	-0.557	0.5781	-0.001
cg24323887	23	HTR2C	0.056	0.026	0.065	0.023	-0.549	0.5833	-0.002
cg19057248	5	CART	0.407	0.032	0.403	0.038	0.536	0.5923	0.002
cg17149245	5	DRD1	0.642	0.049	0.641	0.052	0.534	0.5938	0.003
cg12791151	9	GRIN1	0.289	0.050	0.293	0.056	-0.521	0.6028	-0.004
cg02095443	5	GABRB2	0.069	0.020	0.068	0.018	-0.519	0.6046	-0.001
cg17233601	11	PTPN5	0.318	0.043	0.317	0.047	0.518	0.6053	0.003
cg11155145	4	GABRA4	0.120	0.033	0.122	0.036	0.515	0.6068	0.002
cg16219603	8	PENK	0.079	0.022	0.077	0.019	-0.513	0.6082	-0.001
cg01732192	9	GRIN1	0.034	0.012	0.033	0.014	-0.504	0.6149	-0.001
cg12603785	6	HTR1B	0.201	0.041	0.196	0.037	0.484	0.6285	0.002
cg23623863	23	HTR2C	0.338	0.066	0.308	0.068	-0.476	0.6346	-0.004
cg23210485	9	GRIN1	0.107	0.031	0.108	0.025	-0.472	0.6373	-0.001
cg04915842	11	ANKK1	0.907	0.026	0.910	0.027	-0.462	0.6444	-0.001
cg01449287	2	POMC	0.009	0.008	0.010	0.009	-0.457	0.6479	0.000
cg23806474	20	CHRNA4	0.143	0.023	0.141	0.024	0.457	0.6481	0.001
cg24377504	20	OPRL1	0.039	0.020	0.038	0.015	0.450	0.6530	0.001
cg16514318	1	OPRD1	0.327	0.054	0.332	0.051	0.450	0.6533	0.003

cg02889910	3	SLC6A1	0.133	0.028	0.134	0.026	-0.445	0.6567	-0.001
cg16938887	11	TTC12	0.009	0.024	0.007	0.010	-0.445	0.6569	-0.001
cg00161794	17	PPP1R9B	0.029	0.013	0.029	0.010	-0.444	0.6577	-0.001
cg18423960	4	ADH5	0.537	0.042	0.538	0.050	0.443	0.6584	0.002
cg11548648	9	ALDH1A1	0.045	0.015	0.049	0.018	-0.431	0.6670	-0.001
cg01906966	11	HTR3B	0.541	0.041	0.541	0.048	0.418	0.6762	0.002
cg05010363	23	HTR2C	0.184	0.083	0.233	0.079	-0.410	0.6822	-0.003
cg14993952	17	GRIN2C	0.311	0.062	0.307	0.048	0.410	0.6823	0.003
cg24244000	15	GABRG3	0.445	0.048	0.452	0.048	-0.404	0.6869	-0.002
cg00493111	12	GRIN2B	0.009	0.002	0.009	0.003	0.400	0.6893	0.000
cg07763397	15	GABRB3	0.486	0.080	0.486	0.067	0.390	0.6972	0.004
cg06649410	6	OPRM1	0.104	0.027	0.101	0.025	-0.387	0.6991	-0.001
cg05191437	15	CHRNB4	0.009	0.003	0.009	0.003	0.363	0.7168	0.000
cg12011299	4	ADH4	0.094	0.075	0.099	0.086	-0.362	0.7180	-0.004
cg20672496	17	PPP1R9B	0.270	0.035	0.274	0.030	0.361	0.7186	0.001
cg06914402	3	SLC6A1	0.351	0.101	0.337	0.091	0.347	0.7292	0.004
cg14150516	2	CREB1	0.154	0.038	0.156	0.039	-0.345	0.7306	-0.002
cg09577004	8	OPRK1	0.032	0.015	0.031	0.014	-0.343	0.7322	-0.001
cg14727643	9	GRIN1	0.121	0.024	0.121	0.026	-0.335	0.7382	-0.001
cg17525249	15	GABRA5	0.600	0.058	0.599	0.052	0.333	0.7393	0.002
cg15269722	6	FYN	0.051	0.015	0.052	0.012	-0.309	0.7573	0.000
cg11499300	11	ANKK1	0.809	0.070	0.816	0.052	-0.306	0.7601	-0.002
cg18095295	9	DBH	0.168	0.025	0.173	0.026	-0.301	0.7637	-0.001
cg24645221	8	PENK	0.021	0.007	0.020	0.010	0.299	0.7651	0.000
cg16322193	11	DRD2	0.182	0.037	0.184	0.034	0.293	0.7701	0.001
cg07506561	6	HTR1B	0.122	0.037	0.122	0.038	-0.292	0.7708	-0.001
cg02929485	11	DRD4	0.720	0.076	0.723	0.080	0.282	0.7779	0.003
cg02489623	6	OPRM1	0.087	0.031	0.085	0.031	0.281	0.7791	0.001
cg27601580	8	PNOC	0.243	0.062	0.242	0.059	0.276	0.7829	0.002
cg09744251	15	CHRNA3	0.022	0.007	0.021	0.006	0.269	0.7881	0.000
cg00133192	17	SLC6A4	0.618	0.075	0.643	0.069	-0.262	0.7936	-0.002
cg16772518	4	DRD5	0.718	0.067	0.721	0.054	-0.249	0.8037	-0.002

cg18989937	20	RGS19	0.246	0.025	0.247	0.035	0.249	0.8037	0.001
cg00464020	10	PPA1	0.004	0.016	0.002	0.004	0.242	0.8091	0.000
cg00825193	5	SLC6A3	0.081	0.045	0.074	0.043	0.240	0.8104	0.001
cg16493752	5	DRD1	0.352	0.033	0.355	0.036	-0.238	0.8122	-0.001
cg19303308	4	GABRA4	0.069	0.026	0.074	0.026	-0.230	0.8186	-0.001
cg10550369	15	CHRNB4	0.259	0.027	0.259	0.027	0.222	0.8248	0.001
cg24642067	5	GABRG2	0.043	0.023	0.042	0.016	-0.221	0.8256	-0.001
cg04719837	6	OPRM1	0.074	0.018	0.072	0.016	0.207	0.8363	0.000
cg26000542	23	MAOB	0.019	0.007	0.022	0.006	-0.206	0.8372	0.000
cg14471429	15	GABRB3	0.063	0.020	0.063	0.015	0.202	0.8397	0.000
cg18974026	1	OPRD1	0.470	0.098	0.478	0.098	0.196	0.8446	0.003
cg26409348	4	ADH4	0.437	0.053	0.441	0.053	-0.192	0.8480	-0.001
cg26818805	8	PENK	0.029	0.014	0.027	0.012	0.179	0.8578	0.000
cg10959984	12	ALDH2	0.011	0.004	0.011	0.004	0.171	0.8647	0.000
cg08754277	10	GAD2	0.193	0.027	0.194	0.025	-0.165	0.8689	0.000
cg16570223	15	CHRNA3	0.254	0.027	0.259	0.026	-0.135	0.8929	0.000
cg19473239	8	PENK	0.158	0.062	0.150	0.072	-0.132	0.8950	-0.001
cg21235334	20	DNMT3B	0.217	0.039	0.211	0.031	-0.131	0.8961	-0.001
cg16734072	8	OPRK1	0.116	0.030	0.115	0.028	-0.121	0.9041	0.000
cg11229290	13	HTR2A	0.899	0.040	0.905	0.051	0.111	0.9113	0.001
cg23359453	6	CNR1	0.899	0.030	0.900	0.034	0.110	0.9124	0.000
cg10385651	8	PENK	0.137	0.028	0.129	0.034	0.110	0.9125	0.000
cg00669076	5	HTR1A	0.059	0.015	0.055	0.015	-0.102	0.9191	0.000
cg24172264	20	CHRNA4	0.247	0.053	0.257	0.054	-0.076	0.9392	-0.001
cg00220369	5	GABRA6	0.526	0.043	0.529	0.056	0.073	0.9417	0.000
cg17449649	15	GABRB3	0.059	0.021	0.058	0.020	0.068	0.9456	0.000
cg13985527	8	PNOC	0.657	0.072	0.662	0.072	-0.065	0.9486	-0.001
cg12676896	5	GABRA6	0.741	0.044	0.741	0.050	-0.061	0.9512	0.000
cg06840457	23	HTR2C	0.011	0.005	0.012	0.004	0.056	0.9557	0.000
cg18031916	4	GABRG1	0.385	0.087	0.370	0.097	-0.054	0.9566	-0.001
cg15246991	19	GRIN2D	0.042	0.038	0.039	0.012	-0.046	0.9630	0.000
cg22224310	18	MBD2	0.076	0.029	0.078	0.025	-0.027	0.9788	0.000

cg21248332	10	GAD2	0.055	0.015	0.055	0.016	0.018	0.9857	0.000
cg24260762	6	FYN	0.105	0.019	0.104	0.021	-0.017	0.9863	0.000
cg05578260	15	CHRNA7	0.010	0.004	0.010	0.004	0.012	0.9903	0.000
cg01187684	5	DRD1	0.069	0.058	0.070	0.053	-0.011	0.9916	0.000

Table S2. Association of 384 Promoter CpGs (in 82 genes) and alcohol-nicotine codependence in European Americans (EAs).
(CpGs in red had nominally significant associations with AD-ND codependence in both AAs and EAs)

CpGs	Chr.	Genes	Mean β (Cases)	S.D. (Cases)	Mean β (Controls)	S.D. (Controls)	T statistic	*P* value	Effect size
cg21372728	19	MBD3	0.055	1.459E-02	0.048	0.013	6.135	0.000	0.008
cg27531267	2	HTR2B	0.041	1.141E-02	0.035	0.012	5.406	0.000	0.006
cg26106216	8	PENK	0.031	1.485E-02	0.024	0.012	5.196	0.000	0.007
cg05421426	11	DRD2	0.093	2.125E-02	0.088	0.022	4.762	0.000	0.008
cg14313206	11	NCAM1	0.051	1.093E-02	0.047	0.013	4.612	0.000	0.005
cg02156408	23	HTR2C	0.091	2.233E-02	0.085	0.023	4.605	0.000	0.009
cg21572351	11	NCAM1	0.145	1.666E-02	0.137	0.021	4.598	0.000	0.008
cg08989585	11	HTR3A	0.173	1.492E-02	0.167	0.016	4.452	0.000	0.007
cg02095443	5	GABRB2	0.063	1.608E-02	0.059	0.016	4.229	0.000	0.006
cg01706569	1	OPRD1	0.048	1.554E-02	0.042	0.015	4.199	0.000	0.007
cg08079114	11	DRD4	0.056	1.225E-02	0.051	0.012	4.184	0.000	0.006
cg12505522	6	RGS17	0.059	1.628E-02	0.053	0.018	4.085	0.000	0.007
cg14534584	17	SLC6A4	0.057	1.721E-02	0.051	0.016	4.036	0.000	0.007
cg00037218	5	SLC6A3	0.035	1.386E-02	0.030	0.014	4.034	0.000	0.006
cg07344165	8	OPRK1	0.036	1.511E-02	0.032	0.015	4.012	0.000	0.006
cg04123893	2	GAD1	0.301	2.929E-02	0.292	0.034	3.995	0.000	0.012
cg21074850	4	GABRB1	0.061	1.875E-02	0.056	0.018	3.915	0.000	0.007
cg16024485	20	OPRL1	0.032	1.084E-02	0.028	0.011	3.843	0.000	0.005
cg17610361	4	ADH5	0.086	2.507E-02	0.081	0.026	3.781	0.000	0.009
cg07283003	6	HTR1B	0.788	2.507E-02	0.778	0.029	3.756	0.000	0.013
cg12570007	4	ADH5	0.029	1.198E-02	0.025	0.012	3.747	0.000	0.005
cg16941825	4	DRD5	0.032	1.281E-02	0.028	0.013	3.715	0.000	0.005
cg17987474	15	CHRNA3	0.033	2.103E-02	0.028	0.018	3.704	0.000	0.007
cg10390074	18	MBD1	0.009	2.190E-03	0.009	0.002	3.689	0.000	0.001
cg01768936	11	PTPN5	0.216	2.009E-02	0.209	0.021	3.631	0.000	0.007
cg03896970	20	OPRL1	0.033	1.119E-02	0.031	0.012	3.631	0.000	0.004
cg07975378	17	PPP1R1B	0.046	1.279E-02	0.042	0.012	3.621	0.000	0.005
cg05964444	17	SLC6A4	0.066	1.695E-02	0.060	0.015	3.617	0.000	0.006

cg23759848	5	HTR1A	0.110	2.660E-02	0.106	0.029	3.552	0.000	0.008
cg14544856	2	CREB1	0.040	1.026E-02	0.037	0.010	3.531	0.001	0.003
cg17771682	15	GABRB3	0.015	1.026E-02	0.011	0.010	3.507	0.001	0.004
cg01668758	2	CREB1	0.134	2.523E-02	0.127	0.026	3.506	0.001	0.009
cg26187884	10	GAD2	0.011	7.185E-03	0.009	0.006	3.486	0.001	0.003
cg05884848	17	GRIN2C	0.019	1.316E-02	0.015	0.011	3.405	0.001	0.004
cg25483501	15	CHRNA5	0.080	2.248E-02	0.073	0.018	3.399	0.001	0.008
cg26647484	2	DNMT3A	0.020	7.131E-03	0.017	0.008	3.385	0.001	0.003
cg22646454	5	HTR1A	0.029	1.154E-02	0.026	0.011	3.378	0.001	0.004
cg12966714	19	MBD3	0.029	1.382E-02	0.025	0.012	3.307	0.001	0.004
cg23155627	11	PTPN5	0.050	1.235E-02	0.047	0.014	3.300	0.001	0.004
cg06281629	19	MBD3	0.009	3.511E-03	0.008	0.003	3.290	0.001	0.001
cg01884662	15	CHRNA7	0.113	2.138E-02	0.107	0.024	3.266	0.001	0.007
cg19590658	11	ANKK1	0.075	1.724E-02	0.071	0.019	3.265	0.001	0.006
cg26524348	17	PPP1R9B	0.024	1.088E-02	0.021	0.011	3.204	0.002	0.004
cg22089561	2	GAD1	0.060	1.193E-02	0.056	0.012	3.204	0.002	0.004
cg03109047	9	GRIN1	0.018	8.183E-03	0.014	0.009	3.201	0.002	0.004
cg21590372	12	GRIN2B	0.038	8.165E-03	0.034	0.009	3.177	0.002	0.004
cg10550369	15	CHRNB4	0.269	2.079E-02	0.261	0.023	3.175	0.002	0.008
cg02567788	10	PPA1	0.025	7.606E-03	0.023	0.008	3.141	0.002	0.003
cg11530112	6	CNR1	0.693	1.049E-01	0.728	0.117	-3.120	0.002	-0.045
cg01677874	5	GABRB2	0.105	1.680E-02	0.101	0.018	3.112	0.002	0.005
cg06652524	19	GRIN2D	0.161	2.305E-02	0.153	0.023	3.112	0.002	0.008
cg00243951	11	DRD2	0.012	4.173E-03	0.010	0.005	3.095	0.002	0.002
cg19130396	7	CHRM2	0.067	1.482E-02	0.061	0.015	3.088	0.002	0.006
cg24723845	6	FYN	0.075	9.537E-03	0.072	0.010	3.051	0.003	0.004
cg17466970	2	GAD1	0.039	1.231E-02	0.035	0.014	3.031	0.003	0.004
cg19713819	10	GAD2	0.075	1.974E-02	0.070	0.021	3.015	0.003	0.006
cg19018954	19	DNMT1	0.019	1.197E-02	0.016	0.012	3.002	0.003	0.004
cg19518651	5	GABRA1	0.009	2.624E-03	0.008	0.003	3.001	0.003	0.001
cg12296643	6	FYN	0.043	1.331E-02	0.039	0.012	2.981	0.003	0.004
cg24972720	7	CHRM2	0.030	1.379E-02	0.027	0.014	2.978	0.003	0.004

cg25195998	11	DRD2	0.052	1.557E-02	0.048	0.016	2.977	0.003	0.005
cg05931924	4	DRD5	0.027	1.427E-02	0.024	0.015	2.975	0.003	0.004
cg22872776	20	OPRL1	0.021	1.097E-02	0.018	0.011	2.971	0.003	0.003
cg04719837	6	OPRM1	0.074	1.547E-02	0.069	0.016	2.971	0.003	0.005
cg09864658	9	GRIN1	0.084	2.426E-02	0.076	0.021	2.951	0.004	0.009
cg19289837	2	GAD1	0.026	1.170E-02	0.023	0.012	2.947	0.004	0.004
cg25997474	4	ADH1C	0.069	1.602E-02	0.078	0.024	-2.928	0.004	-0.008
cg18095295	9	DBH	0.179	2.905E-02	0.171	0.029	2.927	0.004	0.010
cg20467969	4	GABRA2	0.079	1.645E-02	0.075	0.016	2.919	0.004	0.005
cg02585344	16	GRIN2A	0.039	1.307E-02	0.036	0.014	2.904	0.004	0.004
cg26300748	5	HTR1A	0.029	1.538E-02	0.024	0.014	2.895	0.004	0.005
cg24774208	10	PPA1	0.016	9.649E-03	0.013	0.009	2.895	0.004	0.003
cg27321505	8	PENK	0.031	1.010E-02	0.028	0.012	2.876	0.005	0.003
cg15234319	15	GABRB3	0.241	5.863E-02	0.256	0.074	-2.869	0.005	-0.021
cg01006616	9	DBH	0.693	3.797E-02	0.682	0.042	2.836	0.005	0.013
cg05286097	17	SLC6A4	0.190	2.571E-02	0.182	0.029	2.800	0.006	0.009
cg08684483	2	GAD1	0.013	6.236E-03	0.011	0.006	2.785	0.006	0.002
cg22851911	3	MBD4	0.098	1.863E-02	0.092	0.018	2.769	0.006	0.006
cg17108064	15	CHRNA5	0.055	1.888E-02	0.048	0.017	2.766	0.006	0.007
cg16164153	20	DNMT3B	0.010	5.686E-03	0.008	0.005	2.757	0.006	0.002
cg03061563	2	HTR2B	0.868	3.787E-02	0.852	0.051	2.748	0.007	0.014
cg22848316	15	CHRNA3	0.024	1.211E-02	0.022	0.012	2.733	0.007	0.003
cg16570223	15	CHRNA3	0.263	2.000E-02	0.257	0.024	2.731	0.007	0.007
cg24591506	16	GRIN2A	0.026	1.129E-02	0.023	0.011	2.731	0.007	0.003
cg00001938	17	GRIN2C	0.026	1.542E-02	0.023	0.016	2.711	0.007	0.004
cg11500467	10	GAD2	0.017	7.569E-03	0.015	0.007	2.670	0.008	0.002
cg06365654	20	CHRNA4	0.019	9.390E-03	0.017	0.008	2.669	0.008	0.003
cg19126369	2	GAD1	0.027	1.305E-02	0.024	0.013	2.658	0.009	0.004
cg10592946	15	CHRNA7	0.093	2.437E-02	0.091	0.024	2.655	0.009	0.006
cg20346122	3	MBD4	0.106	1.593E-02	0.103	0.016	2.654	0.009	0.004
cg26237037	6	RGS17	0.026	9.404E-03	0.022	0.011	2.650	0.009	0.004
cg20650766	15	CHRNA3	0.038	1.240E-02	0.036	0.012	2.646	0.009	0.003

cg00161794	17	PPP1R9B	0.029	9.146E-03	0.028	0.009	2.632	0.009	0.002
cg15291052	15	CHRNA7	0.033	1.197E-02	0.030	0.013	2.628	0.009	0.003
cg22196019	22	COMT	0.008	4.380E-03	0.007	0.004	2.625	0.009	0.001
cg24462132	11	TTC12	0.040	1.057E-02	0.037	0.012	2.615	0.010	0.003
cg13245417	11	PTPN5	0.008	4.012E-03	0.007	0.004	2.609	0.010	0.001
cg07170180	20	DNMT3B	0.040	1.187E-02	0.037	0.013	2.599	0.010	0.004
cg01690182	15	GABRB3	0.140	1.812E-02	0.137	0.018	2.589	0.010	0.004
cg02577095	2	POMC	0.017	1.035E-02	0.015	0.009	2.581	0.011	0.003
cg03476087	10	GAD2	0.020	1.187E-02	0.017	0.011	2.576	0.011	0.003
cg06671711	8	PENK	0.014	8.185E-03	0.011	0.009	2.574	0.011	0.003
cg23137284	15	CHRNA5	0.092	1.837E-02	0.088	0.019	2.573	0.011	0.005
cg00464020	10	PPA1	0.001	2.802E-03	0.003	0.003	-2.555	0.011	-0.001
cg18219951	5	GABRG2	0.029	1.225E-02	0.026	0.012	2.554	0.011	0.003
cg07316621	11	NCAM1	0.011	5.898E-03	0.010	0.006	2.552	0.012	0.002
cg02945241	19	GRIN2D	0.061	1.611E-02	0.059	0.017	2.533	0.012	0.004
cg13887561	6	OPRM1	0.030	1.000E-02	0.027	0.011	2.529	0.012	0.003
cg04550775	12	GRIN2B	0.009	3.257E-03	0.008	0.003	2.520	0.013	0.001
cg16514318	1	OPRD1	0.348	3.890E-02	0.337	0.053	2.518	0.013	0.013
cg01732192	9	GRIN1	0.031	1.222E-02	0.028	0.012	2.503	0.013	0.003
cg15587034	6	HTR1B	0.010	4.070E-03	0.008	0.004	2.491	0.014	0.001
cg01840162	8	OPRK1	0.068	2.243E-02	0.061	0.017	2.478	0.014	0.006
cg16734072	8	OPRK1	0.116	2.496E-02	0.112	0.030	2.477	0.014	0.005
cg20203806	11	DRD2	0.126	2.395E-02	0.124	0.025	2.448	0.015	0.005
cg15244006	20	OPRL1	0.062	1.374E-02	0.060	0.015	2.447	0.015	0.003
cg10615371	15	CHRNA5	0.145	3.280E-02	0.134	0.034	2.377	0.019	0.011
cg16740031	5	HTR1A	0.266	2.615E-02	0.262	0.028	2.359	0.019	0.006
cg11521282	15	CHRNA5	0.015	4.684E-03	0.014	0.005	2.340	0.020	0.001
cg08754277	10	GAD2	0.197	2.205E-02	0.191	0.021	2.300	0.023	0.006
cg02651732	5	SLC6A3	0.030	9.652E-03	0.029	0.011	2.286	0.023	0.002
cg13890276	5	HTR1A	0.046	1.615E-02	0.042	0.017	2.281	0.024	0.005
cg24260762	6	FYN	0.104	1.983E-02	0.102	0.021	2.279	0.024	0.004
cg04915842	11	ANKK1	0.911	1.942E-02	0.904	0.025	2.263	0.025	0.007

cg16219603	8	PENK	0.078	1.786E-02	0.073	0.018	2.262	0.025	0.005
cg17659879	22	MAPK1	0.017	5.691E-03	0.016	0.006	2.257	0.025	0.002
cg12216825	6	HTR1B	0.013	5.373E-03	0.011	0.006	2.228	0.027	0.002
cg16755630	4	GABRB1	0.209	2.397E-02	0.204	0.025	2.228	0.027	0.006
cg18584905	17	SLC6A4	0.139	4.473E-02	0.135	0.044	2.226	0.027	0.008
cg23617770	20	PDYN	0.533	7.697E-02	0.509	0.076	2.224	0.027	0.024
cg01702186	19	GRIN2D	0.023	9.528E-03	0.021	0.010	2.223	0.028	0.002
cg03461962	6	RGS17	0.014	5.769E-03	0.013	0.005	2.217	0.028	0.002
cg20518314	2	DNMT3A	0.015	8.889E-03	0.013	0.009	2.211	0.028	0.002
cg11829658	20	CHRNA4	0.486	9.840E-02	0.463	0.091	2.209	0.028	0.023
cg11861961	5	SLC6A3	0.026	1.130E-02	0.024	0.011	2.197	0.029	0.003
cg21272636	11	TPH1	0.875	3.385E-02	0.865	0.041	2.191	0.030	0.011
cg10400239	20	PDYN	0.332	5.767E-02	0.322	0.060	2.167	0.032	0.010
cg11229290	13	HTR2A	0.910	3.199E-02	0.900	0.048	2.148	0.033	0.012
cg25304536	6	HTR1B	0.057	1.429E-02	0.055	0.014	2.131	0.035	0.003
cg09200586	19	GRIN2D	0.041	1.219E-02	0.040	0.011	2.125	0.035	0.002
cg05807600	4	GABRA2	0.105	2.042E-02	0.102	0.021	2.090	0.038	0.004
cg15268261	13	HTR2A	0.409	3.214E-02	0.406	0.036	2.088	0.038	0.007
cg11227734	2	POMC	0.054	1.400E-02	0.051	0.015	2.074	0.040	0.003
cg26000542	23	MAOB	0.019	6.367E-03	0.018	0.006	2.073	0.040	0.001
cg06319384	15	CHRNB4	0.081	1.586E-02	0.078	0.017	2.070	0.040	0.004
cg18574886	3	MBD4	0.012	6.216E-03	0.011	0.007	2.060	0.041	0.002
cg20573040	3	SLC6A1	0.023	6.504E-03	0.022	0.007	2.030	0.044	0.002
cg24799561	5	GABRA1	0.016	4.654E-03	0.015	0.005	2.030	0.044	0.001
cg00112517	17	PPP1R1B	0.025	1.070E-02	0.023	0.011	2.015	0.045	0.002
cg26409348	4	ADH4	0.453	3.893E-02	0.444	0.045	2.006	0.046	0.011
cg23869328	6	RGS17	0.011	5.075E-03	0.009	0.004	1.991	0.048	0.001
cg17354190	17	GRIN2C	0.012	7.617E-03	0.011	0.009	1.986	0.049	0.002
cg13752831	10	PPA1	0.022	6.179E-03	0.021	0.006	1.970	0.050	0.001
cg08876474	15	CHRNA5	0.009	2.210E-03	0.008	0.003	1.966	0.051	0.001
cg22917359	22	MAPK1	0.869	3.551E-02	0.861	0.038	1.966	0.051	0.009
cg00293936	2	POMC	0.131	2.594E-02	0.126	0.027	1.956	0.052	0.006

cg11153544	7	DDC	0.064	3.552E-02	0.078	0.052	-1.906	0.058	-0.011
cg17484926	11	PTPN5	0.150	3.312E-02	0.143	0.030	1.904	0.059	0.008
cg22358797	18	MBD2	0.056	1.090E-02	0.055	0.011	1.894	0.060	0.002
cg01732618	4	GABRA4	0.028	6.440E-03	0.027	0.007	1.879	0.062	0.001
cg24244000	15	GABRG3	0.463	3.780E-02	0.455	0.047	1.870	0.063	0.010
cg14109444	3	MBD4	0.064	1.371E-02	0.062	0.014	1.869	0.063	0.003
cg09356486	23	MAOA	0.188	2.149E-02	0.185	0.019	1.865	0.064	0.005
cg20787301	19	GRIN2D	0.468	8.991E-02	0.447	0.088	1.832	0.069	0.020
cg05893218	12	GRIN2B	0.050	1.204E-02	0.048	0.014	1.829	0.069	0.003
cg24285775	4	ADH5	0.089	1.803E-02	0.087	0.019	1.802	0.073	0.003
cg14905768	1	OPRD1	0.019	7.081E-03	0.016	0.010	1.800	0.074	0.002
cg20672496	17	PPP1R9B	0.279	2.487E-02	0.276	0.028	1.795	0.074	0.005
cg23798509	3	SLC6A1	0.006	2.116E-03	0.005	0.003	1.788	0.076	0.001
cg03593419	4	GABRA4	0.136	3.896E-02	0.128	0.033	1.770	0.079	0.009
cg23209660	17	PPP1R9B	0.028	1.065E-02	0.027	0.012	1.765	0.079	0.002
cg22921295	5	DRD1	0.043	1.156E-02	0.042	0.013	1.745	0.083	0.002
cg05876300	23	MAOB	0.024	1.448E-02	0.022	0.015	1.714	0.088	0.003
cg01187684	5	DRD1	0.076	5.345E-02	0.065	0.048	1.694	0.092	0.011
cg05408953	23	MECP2	0.807	4.721E-02	0.803	0.047	1.675	0.096	0.007
cg27643501	12	GRIN2B	0.064	1.394E-02	0.062	0.015	1.672	0.096	0.003
cg08642921	19	DNMT1	0.018	1.008E-02	0.017	0.009	1.670	0.097	0.002
cg19391527	8	PNOC	0.725	4.150E-02	0.716	0.046	1.666	0.097	0.010
cg00618092	2	HTR2B	0.584	4.931E-02	0.575	0.051	1.646	0.102	0.010
cg07506561	6	HTR1B	0.120	3.573E-02	0.114	0.037	1.646	0.102	0.008
cg10466664	5	GABRG2	0.023	7.623E-03	0.024	0.011	-1.646	0.102	-0.002
cg04055210	23	GABRA3	0.027	1.248E-02	0.025	0.014	1.640	0.103	0.002
cg10944175	9	DBH	0.096	2.260E-02	0.093	0.021	1.635	0.104	0.005
cg12511279	18	MBD2	0.815	2.306E-02	0.820	0.025	-1.623	0.106	-0.005
cg14009433	23	MAOA	0.080	4.657E-02	0.081	0.044	1.623	0.106	0.004
cg05989757	22	MAPK1	0.005	4.496E-03	0.004	0.004	1.614	0.108	0.001
cg17388934	18	MBD2	0.010	5.909E-03	0.008	0.006	1.607	0.110	0.001
cg06777434	11	NCAM1	0.009	6.169E-03	0.008	0.006	1.603	0.111	0.001

cg08354950	5	CART	0.021	1.100E-02	0.019	0.009	1.599	0.112	0.002
cg04941592	23	MECP2	0.303	3.141E-02	0.301	0.032	1.590	0.114	0.005
cg11880892	10	PPA1	0.014	2.815E-03	0.014	0.003	1.583	0.115	0.001
cg23806474	20	CHRNA4	0.140	3.959E-02	0.135	0.020	1.577	0.117	0.007
cg06840457	23	HTR2C	0.010	3.085E-03	0.009	0.004	1.547	0.124	0.001
cg11499300	11	ANKK1	0.807	3.936E-02	0.797	0.060	1.544	0.124	0.011
cg17915420	2	POMC	0.295	2.245E-02	0.293	0.026	1.529	0.128	0.004
cg13985527	8	PNOC	0.685	6.475E-02	0.668	0.066	1.516	0.131	0.014
cg00779299	5	DRD1	0.872	3.365E-02	0.864	0.037	1.499	0.136	0.007
cg21248332	10	GAD2	0.053	1.136E-02	0.052	0.012	1.497	0.136	0.002
cg07385443	11	DRD4	0.023	6.282E-03	0.021	0.007	1.482	0.140	0.001
cg11487886	2	POMC	0.398	2.812E-02	0.394	0.029	1.476	0.142	0.005
cg11722044	23	MECP2	0.447	6.891E-02	0.441	0.076	1.455	0.148	0.012
cg15408490	12	ALDH2	0.138	5.894E-02	0.134	0.052	1.454	0.148	0.010
cg21239013	12	ALDH2	0.932	1.803E-02	0.928	0.021	1.450	0.149	0.004
cg08339494	19	DNMT1	0.009	1.483E-02	0.012	0.016	-1.449	0.149	-0.003
cg12065362	11	DRD4	0.218	5.105E-02	0.229	0.052	-1.439	0.152	-0.009
cg12139952	4	GABRG1	0.404	7.512E-02	0.389	0.081	1.414	0.159	0.016
cg06938800	15	CHRNA5	0.099	3.172E-02	0.095	0.033	1.409	0.161	0.006
cg05191437	15	CHRNB4	0.008	2.696E-03	0.008	0.003	1.397	0.164	0.001
cg10670893	17	PPP1R1B	0.246	6.294E-02	0.256	0.074	-1.394	0.165	-0.013
cg03088662	16	GRIN2A	0.012	1.315E-02	0.009	0.010	1.372	0.172	0.002
cg16769226	5	DRD1	0.417	2.638E-02	0.412	0.031	1.359	0.176	0.005
cg17137171	11	TTC12	0.829	3.888E-02	0.837	0.054	-1.350	0.179	-0.008
cg12235279	4	GABRG1	0.715	5.065E-02	0.706	0.060	1.350	0.179	0.011
cg05293338	5	HTR1A	0.251	2.479E-02	0.248	0.029	1.346	0.180	0.004
cg14471429	15	GABRB3	0.063	1.287E-02	0.061	0.015	1.340	0.182	0.002
cg18031916	4	GABRG1	0.384	9.116E-02	0.371	0.090	1.339	0.182	0.017
cg16322193	11	DRD2	0.186	2.894E-02	0.182	0.028	1.328	0.186	0.004
cg26258452	17	SLC6A4	0.191	2.761E-02	0.188	0.027	1.316	0.190	0.003
cg24642067	5	GABRG2	0.037	1.689E-02	0.034	0.013	1.302	0.195	0.003
cg23463131	16	GRIN2A	0.036	1.089E-02	0.034	0.012	1.295	0.197	0.002

cg23404860	11	DRD4	0.218	4.423E-02	0.227	0.050	-1.285	0.200	-0.007
cg22515311	4	DRD5	0.257	3.296E-02	0.258	0.035	-1.281	0.202	-0.006
cg00133192	17	SLC6A4	0.622	6.981E-02	0.612	0.080	1.270	0.206	0.012
cg02489623	6	OPRM1	0.073	2.974E-02	0.068	0.032	1.265	0.207	0.005
cg06020661	13	HTR2A	0.717	7.264E-02	0.700	0.079	1.244	0.215	0.013
cg06711394	5	GABRB2	0.060	3.552E-02	0.056	0.018	1.220	0.224	0.005
cg17525249	15	GABRA5	0.595	4.705E-02	0.584	0.060	1.215	0.226	0.008
cg17128529	20	PDYN	0.450	5.616E-02	0.441	0.056	1.213	0.227	0.009
cg21857413	5	HTR1A	0.038	1.600E-02	0.040	0.015	-1.207	0.229	-0.003
cg10959984	12	ALDH2	0.013	3.668E-03	0.013	0.004	1.202	0.231	0.001
cg23079189	11	PTPN5	0.569	8.148E-02	0.557	0.085	1.193	0.234	0.006
cg25624924	7	CHRM2	0.022	6.874E-03	0.020	0.008	1.190	0.236	0.001
cg22224310	18	MBD2	0.081	2.842E-02	0.076	0.029	1.167	0.245	0.005
cg06914402	3	SLC6A1	0.364	1.109E-01	0.339	0.114	1.159	0.248	0.017
cg06649410	6	OPRM1	0.097	2.263E-02	0.094	0.025	1.159	0.248	0.003
cg02340737	4	GABRA2	0.018	1.275E-02	0.017	0.011	1.156	0.249	0.002
cg20648561	6	HTR1B	0.105	4.454E-02	0.099	0.038	1.138	0.257	0.006
cg08289627	18	MBD1	0.559	5.303E-02	0.551	0.057	1.134	0.258	0.006
cg02889910	3	SLC6A1	0.142	2.350E-02	0.141	0.022	1.129	0.260	0.003
cg15130599	5	DRD1	0.215	2.767E-02	0.218	0.029	-1.126	0.262	-0.004
cg00825193	5	SLC6A3	0.077	4.827E-02	0.065	0.043	1.116	0.266	0.007
cg02225257	15	GABRA5	0.512	6.920E-02	0.502	0.075	1.115	0.266	0.011
cg22498099	15	CHRNA5	0.006	4.008E-03	0.005	0.004	1.113	0.267	0.001
cg00669076	5	HTR1A	0.050	1.572E-02	0.051	0.016	-1.100	0.273	-0.002
cg23691961	4	GABRG1	0.050	3.199E-02	0.046	0.029	1.084	0.280	0.005
cg16493752	5	DRD1	0.360	2.468E-02	0.361	0.030	-1.084	0.280	-0.003
cg02811260	6	HTR1B	0.228	4.618E-02	0.220	0.048	1.081	0.281	0.007
cg27580859	23	MAOA	0.405	4.123E-02	0.401	0.031	1.080	0.281	0.005
cg21235334	20	DNMT3B	0.210	3.369E-02	0.213	0.032	-1.074	0.284	-0.005
cg05784269	8	RGS20	0.279	2.517E-02	0.277	0.027	1.058	0.292	0.003
cg24377504	20	OPRL1	0.034	1.143E-02	0.031	0.012	1.057	0.292	0.002
cg24323887	23	HTR2C	0.050	2.038E-02	0.055	0.029	-1.055	0.293	-0.004

cg17636534	23	HTR2C	0.057	4.166E-02	0.062	0.043	-1.048	0.296	-0.004
cg23193606	4	GABRA4	0.010	3.605E-03	0.010	0.004	1.045	0.298	0.001
cg26927763	5	CART	0.345	6.393E-02	0.339	0.061	1.045	0.298	0.007
cg05788582	18	MBD1	0.667	4.723E-02	0.663	0.047	1.037	0.301	0.005
cg23623863	23	HTR2C	0.333	7.414E-02	0.321	0.069	1.017	0.310	0.009
cg27601580	8	PNOC	0.211	5.598E-02	0.201	0.056	1.006	0.316	0.008
cg19303308	4	GABRA4	0.057	2.250E-02	0.062	0.039	-0.978	0.329	-0.004
cg22719623	6	OPRM1	0.076	1.612E-02	0.077	0.015	-0.972	0.332	-0.002
cg20058896	18	MBD1	0.049	1.310E-02	0.047	0.014	0.971	0.333	0.002
cg19285359	5	CART	0.030	9.513E-03	0.028	0.011	0.943	0.347	0.001
cg04527961	15	CHRNB4	0.023	1.635E-02	0.024	0.017	-0.939	0.349	-0.002
cg07081615	5	SLC6A3	0.078	2.202E-02	0.078	0.021	0.916	0.361	0.002
cg17672157	2	CREB1	0.766	6.015E-02	0.758	0.063	0.915	0.361	0.007
cg15232722	8	OPRK1	0.436	8.430E-02	0.441	0.076	-0.910	0.364	-0.010
cg24164433	9	GRIN1	0.473	4.029E-02	0.466	0.053	0.897	0.371	0.005
cg00314411	20	OPRL1	0.124	3.480E-02	0.130	0.034	-0.894	0.373	-0.004
cg18655110	12	ALDH2	0.231	2.766E-02	0.237	0.029	-0.884	0.378	-0.003
cg05578260	15	CHRNA7	0.009	4.031E-03	0.008	0.004	0.878	0.381	0.000
cg03446957	22	COMT	0.006	6.155E-03	0.005	0.007	0.872	0.384	0.001
cg03490309	20	CHRNA4	0.097	2.258E-02	0.097	0.024	0.871	0.385	0.002
cg11722562	20	OPRL1	0.114	3.233E-02	0.121	0.038	-0.850	0.397	-0.003
cg17929169	6	FYN	0.007	3.797E-03	0.007	0.004	0.847	0.398	0.000
cg05227791	23	MECP2	0.348	2.381E-02	0.347	0.026	0.823	0.412	0.002
cg26041285	17	GRIN2C	0.043	2.689E-02	0.046	0.031	-0.809	0.419	-0.003
cg23359453	6	CNR1	0.902	2.396E-02	0.897	0.027	0.805	0.422	0.003
cg13833700	11	PTPN5	0.010	4.661E-03	0.009	0.005	0.800	0.425	0.001
cg02929485	11	DRD4	0.756	4.517E-02	0.753	0.052	0.794	0.428	0.005
cg12791151	9	GRIN1	0.294	5.420E-02	0.299	0.061	-0.784	0.434	-0.006
cg17449649	15	GABRB3	0.057	1.919E-02	0.055	0.018	0.776	0.439	0.002
cg12215457	6	HTR1B	0.086	1.708E-02	0.084	0.019	0.766	0.445	0.002
cg13941250	12	ALDH2	0.943	8.214E-03	0.943	0.012	-0.765	0.445	-0.001
cg09897477	20	CHRNA4	0.022	1.575E-02	0.023	0.016	-0.763	0.447	-0.002

cg01378239	10	PPA1	0.488	7.220E-02	0.484	0.069	0.757	0.450	0.006
cg12902246	20	RGS19	0.174	2.991E-02	0.173	0.030	0.751	0.454	0.003
cg05010363	23	HTR2C	0.174	8.360E-02	0.173	0.084	0.726	0.469	0.006
cg02928015	9	DBH	0.332	5.678E-02	0.329	0.061	0.714	0.476	0.003
cg19057248	5	CART	0.410	3.291E-02	0.407	0.033	0.713	0.477	0.003
cg17111401	11	DRD4	0.457	7.391E-02	0.453	0.080	0.705	0.482	0.007
cg12676896	5	GABRA6	0.734	4.193E-02	0.737	0.048	-0.704	0.483	-0.004
cg19473239	8	PENK	0.112	7.738E-02	0.117	0.059	-0.699	0.486	-0.007
cg15246991	19	GRIN2D	0.036	1.104E-02	0.035	0.013	0.693	0.489	0.001
cg19135956	15	CHRNB4	0.768	2.283E-02	0.768	0.029	0.682	0.496	0.002
cg20621129	11	HTR3A	0.308	4.916E-02	0.306	0.058	0.682	0.496	0.004
cg16405454	11	ANKK1	0.802	5.418E-02	0.794	0.058	0.682	0.496	0.005
cg00295802	5	SLC6A3	0.464	4.070E-02	0.469	0.046	-0.681	0.497	-0.003
cg06841599	23	HTR2C	0.052	2.307E-02	0.055	0.027	-0.669	0.504	-0.002
cg06031989	6	HTR1B	0.041	1.235E-02	0.041	0.013	-0.663	0.508	-0.001
cg10794519	17	GRIN2C	0.101	2.336E-02	0.099	0.024	0.659	0.511	0.002
cg16938887	11	TTC12	0.007	9.101E-03	0.006	0.007	0.645	0.519	0.001
cg15490013	5	DRD1	0.034	9.308E-03	0.035	0.010	-0.642	0.522	-0.001
cg17820491	6	HTR1B	0.028	1.291E-02	0.029	0.013	-0.636	0.526	-0.001
cg26566103	17	RGS9	0.574	4.115E-02	0.575	0.046	-0.634	0.527	-0.003
cg08754521	8	PENK	0.019	9.593E-03	0.018	0.013	-0.623	0.534	-0.001
cg20494803	4	DRD5	0.358	9.133E-02	0.362	0.091	-0.617	0.538	-0.008
cg01163502	1	OPRD1	0.692	5.377E-02	0.697	0.060	-0.611	0.542	-0.005
cg00331892	6	FYN	0.032	1.431E-02	0.031	0.020	0.609	0.544	0.001
cg18620600	15	CHRNB4	0.750	3.737E-02	0.746	0.042	0.599	0.550	0.003
cg20133817	9	GRIN1	0.075	2.226E-02	0.073	0.020	0.595	0.553	0.002
cg05553976	4	DRD5	0.244	2.680E-02	0.246	0.032	0.593	0.554	0.002
cg01709006	5	GABRG2	0.060	1.884E-02	0.058	0.018	0.586	0.559	0.001
cg02404574	12	GRIN2B	0.012	3.569E-03	0.012	0.004	0.578	0.564	0.000
cg15606313	18	MBD1	0.014	7.595E-03	0.014	0.008	0.568	0.571	0.001
cg18989937	20	RGS19	0.239	2.659E-02	0.237	0.030	0.562	0.575	0.001
cg15244476	17	PPP1R9B	0.113	1.919E-02	0.114	0.021	0.538	0.591	0.001

cg14738521	19	DNMT1	0.011	4.485E-03	0.011	0.005	0.530	0.597	0.000
cg20550533	23	MAOB	0.055	5.129E-02	0.062	0.057	-0.516	0.606	-0.002
cg10385651	8	PENK	0.121	2.568E-02	0.116	0.027	0.509	0.612	0.002
cg17149245	5	DRD1	0.661	4.106E-02	0.658	0.053	0.484	0.629	0.003
cg19491443	23	HTR2C	0.516	6.911E-02	0.510	0.080	0.481	0.631	0.005
cg01928350	6	RGS17	0.113	2.298E-02	0.111	0.023	0.479	0.632	0.002
cg20391608	22	COMT	0.175	4.733E-02	0.178	0.052	0.456	0.649	0.003
cg09577004	8	OPRK1	0.030	1.505E-02	0.030	0.015	0.452	0.652	0.001
cg14150516	2	CREB1	0.154	3.651E-02	0.155	0.036	-0.428	0.669	-0.002
cg20574282	3	MBD4	0.031	7.551E-03	0.032	0.009	-0.411	0.682	0.000
cg14727643	9	GRIN1	0.126	3.427E-02	0.124	0.040	0.410	0.683	0.002
cg25204262	11	DRD4	0.774	5.078E-02	0.769	0.053	0.405	0.686	0.002
cg00164724	20	PDYN	0.525	4.882E-02	0.523	0.055	0.402	0.688	0.003
cg01045241	2	DNMT3A	0.279	2.874E-02	0.282	0.031	-0.394	0.694	-0.002
cg12011299	4	ADH4	0.179	1.129E-01	0.186	0.114	-0.380	0.705	-0.006
cg24172264	20	CHRNA4	0.227	5.565E-02	0.228	0.057	0.377	0.706	0.003
cg10000484	5	GABRG2	0.092	2.665E-02	0.093	0.030	0.377	0.707	0.001
cg24863581	18	MBD1	0.891	3.122E-02	0.893	0.024	-0.370	0.712	-0.001
cg09883163	23	MAOA	0.074	7.737E-02	0.084	0.080	-0.349	0.728	-0.001
cg24645221	8	PENK	0.019	8.630E-03	0.018	0.008	0.341	0.733	0.000
cg01325191	15	CHRNB4	0.894	1.948E-02	0.892	0.022	0.340	0.734	0.001
cg02968741	4	GABRB1	0.006	7.767E-03	0.006	0.006	-0.334	0.739	0.000
cg01579505	17	PPP1R9B	0.038	1.164E-02	0.038	0.013	0.299	0.765	0.000
cg00493111	12	GRIN2B	0.008	1.918E-03	0.008	0.002	0.295	0.768	0.000
cg02395672	6	CNR1	0.447	2.894E-02	0.446	0.034	0.294	0.769	0.001
cg01196743	22	MAPK1	0.008	2.355E-03	0.008	0.003	-0.292	0.770	0.000
cg09488889	20	PDYN	0.883	4.451E-02	0.875	0.054	0.291	0.771	0.002
cg08792950	7	DDC	0.903	1.971E-02	0.902	0.026	0.285	0.776	0.001
cg00848742	2	CREB1	0.775	5.726E-02	0.773	0.059	0.284	0.776	0.002
cg15269722	6	FYN	0.053	1.314E-02	0.053	0.014	0.275	0.784	0.000
cg01449287	2	POMC	0.009	9.230E-03	0.008	0.008	0.268	0.789	0.000
cg23424273	6	HTR1B	0.008	4.899E-03	0.008	0.005	-0.262	0.794	0.000

cg06315187	2	POMC	0.413	2.777E-02	0.414	0.034	-0.255	0.799	-0.001
cg18974026	1	OPRD1	0.453	1.015E-01	0.447	0.099	0.252	0.802	0.003
cg09744251	15	CHRNA3	0.019	5.991E-03	0.019	0.005	-0.245	0.807	0.000
cg00220369	5	GABRA6	0.538	4.186E-02	0.539	0.049	0.243	0.809	0.001
cg23593402	20	CHRNA4	0.572	4.406E-02	0.571	0.045	0.226	0.822	0.001
cg11548648	9	ALDH1A1	0.039	1.135E-02	0.040	0.018	0.225	0.822	0.000
cg12040841	8	PNOC	0.615	1.124E-01	0.607	0.103	0.218	0.827	0.002
cg00582628	8	RGS20	0.474	4.823E-02	0.478	0.052	0.217	0.829	0.001
cg01115034	5	SLC6A3	0.173	4.819E-02	0.170	0.058	0.215	0.830	0.002
cg10236526	8	OPRK1	0.027	2.225E-02	0.024	0.027	0.209	0.835	0.001
cg17259718	9	GRIN1	0.202	5.891E-02	0.200	0.055	-0.199	0.843	-0.002
cg04312520	17	GRIN2C	0.219	2.686E-02	0.222	0.028	0.199	0.843	0.000
cg12338263	23	MAOB	0.663	6.768E-02	0.657	0.076	0.191	0.848	0.002
cg06063362	1	OPRD1	0.309	4.364E-02	0.305	0.042	-0.180	0.858	-0.001
cg16865650	6	OPRM1	0.416	1.105E-01	0.414	0.117	-0.172	0.863	-0.002
cg18423960	4	ADH5	0.550	3.254E-02	0.550	0.039	-0.151	0.880	-0.001
cg23296469	3	SLC6A1	0.038	3.624E-02	0.037	0.040	-0.149	0.881	-0.001
cg16206611	9	ALDH1A1	0.073	1.884E-02	0.074	0.027	-0.147	0.883	0.000
cg08411435	17	PPP1R1B	0.600	6.169E-02	0.605	0.071	-0.143	0.886	-0.001
cg23210485	9	GRIN1	0.107	2.636E-02	0.109	0.030	0.140	0.888	0.000
cg20038036	11	DRD4	0.631	7.357E-02	0.627	0.074	-0.129	0.897	-0.001
cg17494568	4	DRD5	0.194	3.406E-02	0.192	0.036	0.128	0.898	0.001
cg22531992	19	MBD3	0.701	6.001E-02	0.701	0.068	0.128	0.898	0.001
cg15123533	17	GRIN2C	0.255	2.864E-02	0.256	0.030	-0.118	0.906	0.000
cg07763397	15	GABRB3	0.453	5.961E-02	0.453	0.066	0.107	0.915	0.001
cg04000318	8	PNOC	0.286	7.782E-02	0.284	0.090	0.084	0.933	0.001
cg26818805	8	PENK	0.022	9.959E-03	0.021	0.012	0.062	0.951	0.000
cg01906966	11	HTR3B	0.552	2.968E-02	0.551	0.035	-0.043	0.966	0.000
cg17233601	11	PTPN5	0.296	4.449E-02	0.296	0.048	0.036	0.971	0.000
cg14602957	2	DNMT3A	0.629	4.473E-02	0.628	0.056	-0.035	0.973	0.000
cg14993952	17	GRIN2C	0.294	4.407E-02	0.292	0.057	-0.027	0.978	0.000
cg12603785	6	HTR1B	0.191	3.540E-02	0.193	0.039	0.023	0.982	0.000

cg06940827	9	DBH	0.526	4.365E-02	0.530	0.048	0.022	0.982	0.000
cg16772518	4	DRD5	0.711	4.655E-02	0.715	0.041	-0.020	0.984	0.000
cg11155145	4	GABRA4	0.109	4.299E-02	0.108	0.045	0.016	0.987	0.000
cg14334548	11	PTPN5	0.090	2.273E-02	0.089	0.025	-0.003	0.998	0.000

Table S3. Information of 384 CpGs in 82 addiction-related genes.
(GGMAScore: generated using the Illumina Assay Design Tool)

CpGs	Gene	Accession_Id	Version	Chr.	Coordinate	GGMAScore	CpG_Island
cg16514318	OPRD1	NM_000911.3	36.1	1	29009502	0.93	FALSE
cg06063362	OPRD1	NM_000911.3	36.1	1	29011014	0.823	TRUE
cg01706569	OPRD1	NM_000911.3	36.1	1	29011768	0.82	TRUE
cg14905768	OPRD1	NM_000911.3	36.1	1	29011843	0.802	TRUE
cg18974026	OPRD1	NM_000911.3	36.1	1	29012064	0.85	TRUE
cg01163502	OPRD1	NM_000911.3	36.1	1	29012185	0.825	TRUE
cg11487886	POMC	NM_000939.1	36.1	2	25244029	0.882	TRUE
cg06315187	POMC	NM_000939.1	36.1	2	25244419	0.831	TRUE
cg11227734	POMC	NM_000939.1	36.1	2	25244702	0.845	TRUE
cg01449287	POMC	NM_000939.1	36.1	2	25244857	0.897	TRUE
cg00293936	POMC	NM_000939.1	36.1	2	25245009	0.831	TRUE
cg02577095	POMC	NM_000939.1	36.1	2	25245228	0.812	TRUE
cg17915420	POMC	NM_000939.1	36.1	2	25246089	0.954	FALSE
cg20518314	DNMT3A	NM_022552.3	36.1	2	25418829	0.931	TRUE
cg26647484	DNMT3A	NM_022552.3	36.1	2	25419043	0.822	TRUE
cg01045241	DNMT3A	NM_022552.3	36.1	2	25419914	0.913	FALSE
cg14602957	DNMT3A	NM_022552.3	36.1	2	25420142	0.826	FALSE
cg19126369	GAD1	NM_013445.3	36.1	2	171379759	0.884	TRUE
cg22089561	GAD1	NM_013445.3	36.1	2	171380171	0.958	TRUE
cg04123893	GAD1	NM_013445.3	36.1	2	171380892	0.863	TRUE
cg17466970	GAD1	NM_013445.3	36.1	2	171381124	0.902	TRUE
cg08684483	GAD1	NM_013445.3	36.1	2	171381706	0.948	TRUE
cg19289837	GAD1	NM_013445.3	36.1	2	171382135	0.833	TRUE
cg00848742	CREB1	NM_004379.2	36.1	2	208101141	0.93	FALSE
cg17672157	CREB1	NM_004379.2	36.1	2	208101551	0.887	TRUE
cg14544856	CREB1	NM_004379.2	36.1	2	208102228	0.891	TRUE
cg14150516	CREB1	NM_004379.2	36.1	2	208103029	0.944	TRUE
cg01668758	CREB1	NM_004379.2	36.1	2	208103715	0.846	TRUE
cg03061563	HTR2B	NM_000867.2	36.1	2	231698652	0.871	FALSE

cg00618092	HTR2B	NM_000867.2	36.1	2	231699329	0.826	FALSE
cg27531267	HTR2B	NM_000867.2	36.1	2	231699986	0.825	FALSE
cg23798509	SLC6A1	NM_003042.2	36.1	3	11008878	0.829	FALSE
cg20573040	SLC6A1	NM_003042.2	36.1	3	11009247	0.91	TRUE
cg23296469	SLC6A1	NM_003042.2	36.1	3	11009493	0.849	TRUE
cg06914402	SLC6A1	NM_003042.2	36.1	3	11009936	0.846	TRUE
cg02889910	SLC6A1	NM_003042.2	36.1	3	11010147	0.941	TRUE
cg20574282	MBD4	NM_003925.1	36.1	3	130641141	0.952	TRUE
cg20346122	MBD4	NM_003925.1	36.1	3	130641324	0.802	TRUE
cg18574886	MBD4	NM_003925.1	36.1	3	130641734	0.863	TRUE
cg22851911	MBD4	NM_003925.1	36.1	3	130642065	0.853	TRUE
cg14109444	MBD4	NM_003925.1	36.1	3	130642193	0.869	TRUE
cg17494568	DRD5	NM_000798.3	36.1	4	9390759	0.953	FALSE
cg16941825	DRD5	NM_000798.3	36.1	4	9392240	0.813	TRUE
cg05931924	DRD5	NM_000798.3	36.1	4	9392369	0.826	TRUE
cg05553976	DRD5	NM_000798.3	36.1	4	9392699	0.81	TRUE
cg22515311	DRD5	NM_000798.3	36.1	4	9392880	0.824	TRUE
cg20494803	DRD5	NM_000798.3	36.1	4	9392991	0.833	TRUE
cg16772518	DRD5	NM_000798.3	36.1	4	9393353	0.805	TRUE
cg18031916	GABRG1	NM_173536.3	36.1	4	45820704	0.8	FALSE
cg23691961	GABRG1	NM_173536.3	36.1	4	45821130	0.906	TRUE
cg12139952	GABRG1	NM_173536.3	36.1	4	45821970	0.828	FALSE
cg12235279	GABRG1	NM_173536.3	36.1	4	45822093	0.859	FALSE
cg20467969	GABRA2	NM_000807.1	36.1	4	46086119	0.847	TRUE
cg02340737	GABRA2	NM_000807.1	36.1	4	46086402	0.827	TRUE
cg05807600	GABRA2	NM_000807.1	36.1	4	46087619	0.814	FALSE
cg11155145	GABRA4	NM_000809.2	36.1	4	46689583	0.843	FALSE
cg19303308	GABRA4	NM_000809.2	36.1	4	46689658	0.857	FALSE
cg01732618	GABRA4	NM_000809.2	36.1	4	46689934	0.831	TRUE
cg03593419	GABRA4	NM_000809.2	36.1	4	46690231	0.858	TRUE
cg23193606	GABRA4	NM_000809.2	36.1	4	46690496	0.824	TRUE
cg16755630	GABRB1	NM_000812.2	36.1	4	46728031	0.867	FALSE

cg02968741	GABRB1	NM_000812.2	36.1	4	46728180	0.914	TRUE
cg21074850	GABRB1	NM_000812.2	36.1	4	46729246	0.806	TRUE
cg24285775	ADH5	NM_000671.3	36.1	4	100228415	0.81	FALSE
cg17610361	ADH5	NM_000671.3	36.1	4	100228846	0.835	TRUE
cg12570007	ADH5	NM_000671.3	36.1	4	100229045	0.825	TRUE
cg18423960	ADH5	NM_000671.3	36.1	4	100229673	0.83	TRUE
cg12011299	ADH4	NM_000670.3	36.1	4	100284569	0.833	FALSE
cg26409348	ADH4	NM_000670.3	36.1	4	100285646	0.834	FALSE
cg25997474	ADH1C	NM_000669.3	36.1	4	100492992	0.863	FALSE
cg01115034	SLC6A3	NM_001044.2	36.1	5	1497607	0.809	TRUE
cg00825193	SLC6A3	NM_001044.2	36.1	5	1497910	0.821	TRUE
cg00295802	SLC6A3	NM_001044.2	36.1	5	1498084	0.804	TRUE
cg00037218	SLC6A3	NM_001044.2	36.1	5	1498390	0.801	TRUE
cg11861961	SLC6A3	NM_001044.2	36.1	5	1498711	0.952	TRUE
cg07081615	SLC6A3	NM_001044.2	36.1	5	1498835	0.808	TRUE
cg02651732	SLC6A3	NM_001044.2	36.1	5	1499517	0.819	TRUE
cg26300748	HTR1A	NM_000524.2	36.1	5	63292413	0.82	TRUE
cg13890276	HTR1A	NM_000524.2	36.1	5	63292581	0.861	TRUE
cg16740031	HTR1A	NM_000524.2	36.1	5	63292793	0.85	TRUE
cg21857413	HTR1A	NM_000524.2	36.1	5	63293021	0.825	TRUE
cg22646454	HTR1A	NM_000524.2	36.1	5	63293212	0.838	TRUE
cg00669076	HTR1A	NM_000524.2	36.1	5	63293383	0.821	TRUE
cg23759848	HTR1A	NM_000524.2	36.1	5	63293484	0.813	TRUE
cg05293338	HTR1A	NM_000524.2	36.1	5	63294378	0.824	TRUE
cg26927763	CART	NM_004291.2	36.1	5	71050448	0.887	FALSE
cg19285359	CART	NM_004291.2	36.1	5	71050637	0.823	TRUE
cg08354950	CART	NM_004291.2	36.1	5	71051023	0.839	TRUE
cg19057248	CART	NM_004291.2	36.1	5	71051470	0.828	TRUE
cg06711394	GABRB2	NM_021911.1	36.1	5	160907138	0.922	TRUE
cg02095443	GABRB2	NM_021911.1	36.1	5	160908531	0.881	TRUE
cg01677874	GABRB2	NM_021911.1	36.1	5	160908858	0.912	TRUE
cg00220369	GABRA6	NM_000811.1	36.1	5	161045241	0.935	FALSE

cg12676896	GABRA6	NM_000811.1	36.1	5	161045868	0.871	FALSE
cg24799561	GABRA1	NM_000806.3	36.1	5	161207199	0.941	TRUE
cg19518651	GABRA1	NM_000806.3	36.1	5	161207945	0.827	TRUE
cg10000484	GABRG2	NM_000816.2	36.1	5	161427014	0.895	FALSE
cg18219951	GABRG2	NM_000816.2	36.1	5	161427388	0.807	TRUE
cg01709006	GABRG2	NM_000816.2	36.1	5	161427678	0.86	TRUE
cg24642067	GABRG2	NM_000816.2	36.1	5	161428018	0.902	TRUE
cg10466664	GABRG2	NM_000816.2	36.1	5	161428088	0.951	TRUE
cg00779299	DRD1	NM_000794.2	36.1	5	174802870	0.818	FALSE
cg15130599	DRD1	NM_000794.2	36.1	5	174803257	0.816	TRUE
cg15490013	DRD1	NM_000794.2	36.1	5	174803418	0.825	TRUE
cg22921295	DRD1	NM_000794.2	36.1	5	174803673	0.811	TRUE
cg01187684	DRD1	NM_000794.2	36.1	5	174803858	0.888	TRUE
cg16769226	DRD1	NM_000794.2	36.1	5	174804705	0.904	TRUE
cg17149245	DRD1	NM_000794.2	36.1	5	174805530	0.82	TRUE
cg16493752	DRD1	NM_000794.2	36.1	5	174805654	0.815	TRUE
cg17820491	HTR1B	NM_000863.1	36.1	6	78228951	0.903	TRUE
cg15587034	HTR1B	NM_000863.1	36.1	6	78229082	0.813	TRUE
cg25304536	HTR1B	NM_000863.1	36.1	6	78229201	0.947	TRUE
cg12603785	HTR1B	NM_000863.1	36.1	6	78229400	0.829	TRUE
cg12216825	HTR1B	NM_000863.1	36.1	6	78229575	0.914	TRUE
cg23424273	HTR1B	NM_000863.1	36.1	6	78229946	0.938	TRUE
cg12215457	HTR1B	NM_000863.1	36.1	6	78230242	0.928	FALSE
cg20648561	HTR1B	NM_000863.1	36.1	6	78230507	0.805	TRUE
cg06031989	HTR1B	NM_000863.1	36.1	6	78230761	0.872	TRUE
cg07283003	HTR1B	NM_000863.1	36.1	6	78230890	0.853	TRUE
cg07506561	HTR1B	NM_000863.1	36.1	6	78231024	0.834	FALSE
cg02811260	HTR1B	NM_000863.1	36.1	6	78231160	0.809	FALSE
cg23359453	CNR1	NM_016083.3	36.1	6	88910860	0.808	TRUE
cg02395672	CNR1	NM_016083.3	36.1	6	88911005	0.85	TRUE
cg11530112	CNR1	NM_016083.3	36.1	6	88911168	0.801	TRUE
cg00331892	FYN	NM_002037.3	36.1	6	112300691	0.812	TRUE

cg12296643	FYN	NM_002037.3	36.1	6	112300988	0.796	TRUE
cg24260762	FYN	NM_002037.3	36.1	6	112301448	0.811	TRUE
cg24723845	FYN	NM_002037.3	36.1	6	112301815	0.789	FALSE
cg15269722	FYN	NM_002037.3	36.1	6	112302030	0.915	FALSE
cg17929169	FYN	NM_002037.3	36.1	6	112302215	0.7	FALSE
cg26237037	RGS17	NM_012419.4	36.1	6	153493192	0.892	TRUE
cg03461962	RGS17	NM_012419.4	36.1	6	153493474	0.837	TRUE
cg23869328	RGS17	NM_012419.4	36.1	6	153493953	0.896	TRUE
cg12505522	RGS17	NM_012419.4	36.1	6	153494537	0.88	TRUE
cg01928350	RGS17	NM_012419.4	36.1	6	153494766	0.863	TRUE
cg06649410	OPRM1	NM_000914.2	36.1	6	154402176	0.924	TRUE
cg13887561	OPRM1	NM_000914.2	36.1	6	154402323	0.907	TRUE
cg22719623	OPRM1	NM_000914.2	36.1	6	154402425	0.922	TRUE
cg04719837	OPRM1	NM_000914.2	36.1	6	154402578	0.936	TRUE
cg02489623	OPRM1	NM_000914.2	36.1	6	154402700	0.696	TRUE
cg16865650	OPRM1	NM_000914.2	36.1	6	154402801	0.756	TRUE
cg11153544	DDC	NM_000790.2	36.1	7	50595302	0.863	FALSE
cg08792950	DDC	NM_000790.2	36.1	7	50595741	0.827	FALSE
cg24972720	CHRM2	NM_000739.2	36.1	7	136203999	0.805	TRUE
cg19130396	CHRM2	NM_000739.2	36.1	7	136204092	0.836	TRUE
cg25624924	CHRM2	NM_000739.2	36.1	7	136204567	0.954	TRUE
cg19391527	PNOC	NM_006228.2	36.1	8	28229651	0.955	FALSE
cg12040841	PNOC	NM_006228.2	36.1	8	28230553	0.863	FALSE
cg27601580	PNOC	NM_006228.2	36.1	8	28230707	0.716	FALSE
cg04000318	PNOC	NM_006228.2	36.1	8	28231327	0.918	FALSE
cg13985527	PNOC	NM_006228.2	36.1	8	28231544	0.958	FALSE
cg01840162	OPRK1	NM_000912.3	36.1	8	54325831	0.833	TRUE
cg10236526	OPRK1	NM_000912.3	36.1	8	54325917	0.734	TRUE
cg09577004	OPRK1	NM_000912.3	36.1	8	54326093	0.732	TRUE
cg07344165	OPRK1	NM_000912.3	36.1	8	54326330	0.731	TRUE
cg16734072	OPRK1	NM_000912.3	36.1	8	54326599	0.767	TRUE
cg15232722	OPRK1	NM_000912.3	36.1	8	54327916	0.821	FALSE

cg05784269	RGS20	NM_003702.2	36.1	8	54925936	0.826	TRUE
cg00582628	RGS20	NM_003702.2	36.1	8	54927068	0.491	FALSE
cg10385651	PENK	NM_006211.2	36.1	8	57520878	0.846	TRUE
cg26106216	PENK	NM_006211.2	36.1	8	57521167	0.916	TRUE
cg06671711	PENK	NM_006211.2	36.1	8	57521311	0.817	TRUE
cg24645221	PENK	NM_006211.2	36.1	8	57521482	0.815	TRUE
cg27321505	PENK	NM_006211.2	36.1	8	57521713	0.84	TRUE
cg08754521	PENK	NM_006211.2	36.1	8	57522033	0.904	TRUE
cg26818805	PENK	NM_006211.2	36.1	8	57522226	0.816	TRUE
cg19473239	PENK	NM_006211.2	36.1	8	57522492	0.821	TRUE
cg16219603	PENK	NM_006211.2	36.1	8	57523140	0.853	TRUE
cg16206611	ALDH1A1	NM_000689.3	36.1	9	74756981	0.939	FALSE
cg11548648	ALDH1A1	NM_000689.3	36.1	9	74757183	0.876	FALSE
cg18095295	DBH	NM_000787.2	36.1	9	135489518	0.816	FALSE
cg10944175	DBH	NM_000787.2	36.1	9	135490020	0.859	FALSE
cg06940827	DBH	NM_000787.2	36.1	9	135490620	0.883	FALSE
cg02928015	DBH	NM_000787.2	36.1	9	135491188	0.885	FALSE
cg01006616	DBH	NM_000787.2	36.1	9	135491995	0.801	TRUE
cg09864658	GRIN1	NM_021569.1	36.1	9	139150707	0.805	FALSE
cg14727643	GRIN1	NM_021569.1	36.1	9	139150982	0.948	FALSE
cg12791151	GRIN1	NM_021569.1	36.1	9	139151118	0.865	FALSE
cg20133817	GRIN1	NM_021569.1	36.1	9	139151608	0.83	FALSE
cg24164433	GRIN1	NM_021569.1	36.1	9	139151849	0.807	TRUE
cg03109047	GRIN1	NM_021569.1	36.1	9	139152507	0.855	FALSE
cg17259718	GRIN1	NM_021569.1	36.1	9	139152728	0.811	TRUE
cg01732192	GRIN1	NM_021569.1	36.1	9	139153162	0.901	TRUE
cg23210485	GRIN1	NM_021569.1	36.1	9	139153405	0.904	TRUE
cg08754277	GAD2	NM_000818.1	36.1	10	26543784	0.957	TRUE
cg26187884	GAD2	NM_000818.1	36.1	10	26544008	0.813	TRUE
cg11500467	GAD2	NM_000818.1	36.1	10	26544586	0.827	TRUE
cg21248332	GAD2	NM_000818.1	36.1	10	26545324	0.831	TRUE
cg19713819	GAD2	NM_000818.1	36.1	10	26545708	0.893	TRUE

cg03476087	GAD2	NM_000818.1	36.1	10	26546359	0.825	TRUE
cg02567788	PPA1	NM_021129.2	36.1	10	71662285	0.953	TRUE
cg11880892	PPA1	NM_021129.2	36.1	10	71662666	0.888	TRUE
cg24774208	PPA1	NM_021129.2	36.1	10	71663037	0.918	TRUE
cg00464020	PPA1	NM_021129.2	36.1	10	71663169	0.829	TRUE
cg13752831	PPA1	NM_021129.2	36.1	10	71663541	0.891	TRUE
cg01378239	PPA1	NM_021129.2	36.1	10	71664552	0.836	TRUE
cg20038036	DRD4	NM_000797.2	36.1	11	625685	0.94	FALSE
cg25204262	DRD4	NM_000797.2	36.1	11	626156	0.87	FALSE
cg02929485	DRD4	NM_000797.2	36.1	11	626452	0.824	TRUE
cg17111401	DRD4	NM_000797.2	36.1	11	626621	0.84	TRUE
cg07385443	DRD4	NM_000797.2	36.1	11	627570	0.844	TRUE
cg12065362	DRD4	NM_000797.2	36.1	11	627832	0.908	TRUE
cg08079114	DRD4	NM_000797.2	36.1	11	627981	0.835	FALSE
cg23404860	DRD4	NM_000797.2	36.1	11	628245	0.857	TRUE
cg21272636	TPH1	NM_004179.1	36.1	11	18019702	0.877	FALSE
cg14334548	PTPN5	NM_032781.2	36.1	11	18769300	0.869	TRUE
cg13833700	PTPN5	NM_032781.2	36.1	11	18769927	0.81	TRUE
cg17484926	PTPN5	NM_032781.2	36.1	11	18770403	0.807	TRUE
cg13245417	PTPN5	NM_032781.2	36.1	11	18770604	0.905	TRUE
cg23155627	PTPN5	NM_032781.2	36.1	11	18770814	0.912	TRUE
cg17233601	PTPN5	NM_032781.2	36.1	11	18770994	0.888	TRUE
cg23079189	PTPN5	NM_032781.2	36.1	11	18771178	0.909	FALSE
cg01768936	PTPN5	NM_032781.2	36.1	11	18771565	0.916	TRUE
cg21572351	NCAM1	NM_000615.1	36.1	11	112335975	0.836	TRUE
cg14313206	NCAM1	NM_000615.1	36.1	11	112337308	0.843	TRUE
cg06777434	NCAM1	NM_000615.1	36.1	11	112337716	0.834	TRUE
cg07316621	NCAM1	NM_000615.1	36.1	11	112338176	0.809	TRUE
cg16938887	TTC12	NM_017868.2	36.1	11	112690314	0.828	TRUE
cg24462132	TTC12	NM_017868.2	36.1	11	112690625	0.911	TRUE
cg17137171	TTC12	NM_017868.2	36.1	11	112691318	0.874	FALSE
cg04915842	ANKK1	NM_178510.1	36.1	11	112761910	0.902	FALSE

cg16405454	ANKK1	NM_178510.1	36.1	11	112763433	0.838	FALSE
cg19590658	ANKK1	NM_178510.1	36.1	11	112763841	0.869	TRUE
cg11499300	ANKK1	NM_178510.1	36.1	11	112764262	0.908	FALSE
cg20203806	DRD2	NM_016574.2	36.1	11	112850093	0.834	FALSE
cg16322193	DRD2	NM_016574.2	36.1	11	112850362	0.826	TRUE
cg05421426	DRD2	NM_016574.2	36.1	11	112850463	0.833	TRUE
cg25195998	DRD2	NM_016574.2	36.1	11	112850817	0.907	TRUE
cg00243951	DRD2	NM_016574.2	36.1	11	112850934	0.935	TRUE
cg01906966	HTR3B	NM_006028.3	36.1	11	113279424	0.809	FALSE
cg08989585	HTR3A	NM_000869.2	36.1	11	113350239	0.842	FALSE
cg20621129	HTR3A	NM_000869.2	36.1	11	113351214	0.931	FALSE
cg02404574	GRIN2B	NM_000834.2	36.1	12	14024719	0.81	TRUE
cg04550775	GRIN2B	NM_000834.2	36.1	12	14024902	0.841	TRUE
cg27643501	GRIN2B	NM_000834.2	36.1	12	14025056	0.862	TRUE
cg05893218	GRIN2B	NM_000834.2	36.1	12	14025390	0.825	TRUE
cg00493111	GRIN2B	NM_000834.2	36.1	12	14025456	0.943	TRUE
cg21590372	GRIN2B	NM_000834.2	36.1	12	14026300	0.853	TRUE
cg18655110	ALDH2	NM_000690.2	36.1	12	110688136	0.826	TRUE
cg13941250	ALDH2	NM_000690.2	36.1	12	110688272	0.833	TRUE
cg21239013	ALDH2	NM_000690.2	36.1	12	110688555	0.889	TRUE
cg10959984	ALDH2	NM_000690.2	36.1	12	110688763	0.842	TRUE
cg15408490	ALDH2	NM_000690.2	36.1	12	110688974	0.804	TRUE
cg11229290	HTR2A	NM_000621.2	36.1	13	46367888	0.844	FALSE
cg15268261	HTR2A	NM_000621.2	36.1	13	46369029	0.853	FALSE
cg06020661	HTR2A	NM_000621.2	36.1	13	46370139	0.94	TRUE
cg17449649	GABRB3	NM_021912.2	36.1	15	24569555	0.866	TRUE
cg14471429	GABRB3	NM_021912.2	36.1	15	24569644	0.86	TRUE
cg17771682	GABRB3	NM_021912.2	36.1	15	24569866	0.819	TRUE
cg01690182	GABRB3	NM_021912.2	36.1	15	24569967	0.894	TRUE
cg15234319	GABRB3	NM_021912.2	36.1	15	24570486	0.945	FALSE
cg07763397	GABRB3	NM_021912.2	36.1	15	24571958	0.942	FALSE
cg02225257	GABRA5	NM_000810.2	36.1	15	24741680	0.808	FALSE

cg17525249	GABRA5	NM_000810.2	36.1	15	24742740	0.812	FALSE
cg24244000	GABRG3	NM_033223.1	36.1	15	25343930	0.818	FALSE
cg01884662	CHRNA7	NM_000746.2	36.1	15	30109439	0.915	TRUE
cg15291052	CHRNA7	NM_000746.2	36.1	15	30109552	0.822	TRUE
cg05578260	CHRNA7	NM_000746.2	36.1	15	30109702	0.9	TRUE
cg10592946	CHRNA7	NM_000746.2	36.1	15	30110163	0.803	TRUE
cg17108064	CHRNA5	NM_000745.2	36.1	15	76644115	0.809	TRUE
cg10615371	CHRNA5	NM_000745.2	36.1	15	76644210	0.826	TRUE
cg22498099	CHRNA5	NM_000745.2	36.1	15	76644626	0.814	FALSE
cg06938800	CHRNA5	NM_000745.2	36.1	15	76644836	0.925	TRUE
cg08876474	CHRNA5	NM_000745.2	36.1	15	76645023	0.851	TRUE
cg23137284	CHRNA5	NM_000745.2	36.1	15	76645276	0.918	TRUE
cg25483501	CHRNA5	NM_000745.2	36.1	15	76645443	0.826	TRUE
cg11521282	CHRNA5	NM_000745.2	36.1	15	76645767	0.847	FALSE
cg20650766	CHRNA3	NM_000743.2	36.1	15	76699527	0.803	TRUE
cg22848316	CHRNA3	NM_000743.2	36.1	15	76700177	0.887	TRUE
cg17987474	CHRNA3	NM_000743.2	36.1	15	76700367	0.884	TRUE
cg09744251	CHRNA3	NM_000743.2	36.1	15	76700606	0.811	TRUE
cg16570223	CHRNA3	NM_000743.2	36.1	15	76701494	0.815	FALSE
cg19135956	CHRNB4	NM_000750.2	36.1	15	76719822	0.857	FALSE
cg10550369	CHRNB4	NM_000750.2	36.1	15	76720249	0.841	FALSE
cg05191437	CHRNB4	NM_000750.2	36.1	15	76720491	0.911	TRUE
cg06319384	CHRNB4	NM_000750.2	36.1	15	76720640	0.846	TRUE
cg04527961	CHRNB4	NM_000750.2	36.1	15	76720756	0.959	TRUE
cg01325191	CHRNB4	NM_000750.2	36.1	15	76721233	0.946	FALSE
cg18620600	CHRNB4	NM_000750.2	36.1	15	76721875	0.935	FALSE
cg24591506	GRIN2A	NM_000833.2	36.1	16	10183405	0.814	TRUE
cg02585344	GRIN2A	NM_000833.2	36.1	16	10183593	0.865	FALSE
cg23463131	GRIN2A	NM_000833.2	36.1	16	10183911	0.825	TRUE
cg03088662	GRIN2A	NM_000833.2	36.1	16	10184420	0.929	TRUE
cg00133192	SLC6A4	NM_001045.2	36.1	17	25586147	0.847	FALSE
cg05286097	SLC6A4	NM_001045.2	36.1	17	25586390	0.816	TRUE

cg26258452	SLC6A4	NM_001045.2	36.1	17	25586591	0.905	TRUE
cg14534584	SLC6A4	NM_001045.2	36.1	17	25587232	0.809	TRUE
cg18584905	SLC6A4	NM_001045.2	36.1	17	25587426	0.969	FALSE
cg05964444	SLC6A4	NM_001045.2	36.1	17	25588133	0.834	TRUE
cg10670893	PPP1R1B	NM_032192.2	36.1	17	35036023	0.82	FALSE
cg07975378	PPP1R1B	NM_032192.2	36.1	17	35036257	0.804	FALSE
cg00112517	PPP1R1B	NM_032192.2	36.1	17	35036537	0.808	FALSE
cg08411435	PPP1R1B	NM_032192.2	36.1	17	35037550	0.863	TRUE
cg20672496	PPP1R9B	NM_032595.1	36.1	17	45581875	0.831	TRUE
cg01579505	PPP1R9B	NM_032595.1	36.1	17	45582155	0.827	TRUE
cg00161794	PPP1R9B	NM_032595.1	36.1	17	45582549	0.82	TRUE
cg15244476	PPP1R9B	NM_032595.1	36.1	17	45582714	0.832	TRUE
cg26524348	PPP1R9B	NM_032595.1	36.1	17	45583444	0.813	TRUE
cg23209660	PPP1R9B	NM_032595.1	36.1	17	45584207	0.879	FALSE
cg26566103	RGS9	NM_003835.1	36.1	17	60614977	0.774	FALSE
cg14993952	GRIN2C	NM_000835.3	36.1	17	70367117	0.946	TRUE
cg17354190	GRIN2C	NM_000835.3	36.1	17	70367659	0.809	TRUE
cg10794519	GRIN2C	NM_000835.3	36.1	17	70367862	0.82	TRUE
cg15123533	GRIN2C	NM_000835.3	36.1	17	70368359	0.848	TRUE
cg26041285	GRIN2C	NM_000835.3	36.1	17	70368724	0.883	TRUE
cg00001938	GRIN2C	NM_000835.3	36.1	17	70369041	0.818	TRUE
cg05884848	GRIN2C	NM_000835.3	36.1	17	70369423	0.828	TRUE
cg04312520	GRIN2C	NM_000835.3	36.1	17	70369601	0.897	TRUE
cg20058896	MBD1	NM_015845.2	36.1	18	46061273	0.982	FALSE
cg10390074	MBD1	NM_015845.2	36.1	18	46061476	0.911	TRUE
cg15606313	MBD1	NM_015845.2	36.1	18	46062070	0.854	TRUE
cg24863581	MBD1	NM_015845.2	36.1	18	46062563	0.927	TRUE
cg08289627	MBD1	NM_015845.2	36.1	18	46062869	0.816	TRUE
cg05788582	MBD1	NM_015845.2	36.1	18	46063280	0.826	TRUE
cg22358797	MBD2	NM_003927.3	36.1	18	50004186	0.986	TRUE
cg17388934	MBD2	NM_003927.3	36.1	18	50005110	0.833	TRUE
cg22224310	MBD2	NM_003927.3	36.1	18	50005261	0.811	TRUE

cg12511279	MBD2	NM_003927.3	36.1	18	50006567	0.819	TRUE
cg12966714	MBD3	NM_003926.5	36.1	19	1542898	0.879	TRUE
cg21372728	MBD3	NM_003926.5	36.1	19	1543111	0.805	TRUE
cg06281629	MBD3	NM_003926.5	36.1	19	1544215	0.885	TRUE
cg22531992	MBD3	NM_003926.5	36.1	19	1544803	0.946	FALSE
cg19018954	DNMT1	NM_001379.1	36.1	19	10166090	0.808	TRUE
cg08642921	DNMT1	NM_001379.1	36.1	19	10166280	0.814	TRUE
cg08339494	DNMT1	NM_001379.1	36.1	19	10166472	0.846	TRUE
cg14738521	DNMT1	NM_001379.1	36.1	19	10166724	0.84	TRUE
cg20787301	GRIN2D	NM_000836.1	36.1	19	53588165	0.809	FALSE
cg06652524	GRIN2D	NM_000836.1	36.1	19	53588716	0.877	TRUE
cg02945241	GRIN2D	NM_000836.1	36.1	19	53588921	0.812	TRUE
cg15246991	GRIN2D	NM_000836.1	36.1	19	53589330	0.898	FALSE
cg01702186	GRIN2D	NM_000836.1	36.1	19	53589710	0.805	FALSE
cg09200586	GRIN2D	NM_000836.1	36.1	19	53589855	0.829	TRUE
cg10400239	PDYN	NM_024411.2	36.1	20	1922045	0.74	FALSE
cg23617770	PDYN	NM_024411.2	36.1	20	1922524	0.894	FALSE
cg09488889	PDYN	NM_024411.2	36.1	20	1923325	0.899	TRUE
cg00164724	PDYN	NM_024411.2	36.1	20	1923406	0.839	TRUE
cg17128529	PDYN	NM_024411.2	36.1	20	1923518	0.604	FALSE
cg16164153	DNMT3B	NM_006892.3	36.1	20	30814431	0.916	TRUE
cg07170180	DNMT3B	NM_006892.3	36.1	20	30814651	0.884	TRUE
cg21235334	DNMT3B	NM_006892.3	36.1	20	30814832	0.821	TRUE
cg03490309	CHRNA4	NM_000744.2	36.1	20	61462346	0.818	FALSE
cg09897477	CHRNA4	NM_000744.2	36.1	20	61463520	0.813	TRUE
cg06365654	CHRNA4	NM_000744.2	36.1	20	61463755	0.834	TRUE
cg23806474	CHRNA4	NM_000744.2	36.1	20	61464027	0.842	TRUE
cg24172264	CHRNA4	NM_000744.2	36.1	20	61464272	0.85	FALSE
cg23593402	CHRNA4	NM_000744.2	36.1	20	61464379	0.942	FALSE
cg11829658	CHRNA4	NM_000744.2	36.1	20	61464528	0.852	FALSE
cg22872776	OPRL1	NM_182647.1	36.1	20	62181094	0.851	FALSE
cg11722562	OPRL1	NM_182647.1	36.1	20	62181521	0.847	TRUE

cg15244006	OPRL1	NM_182647.1	36.1	20	62181926	0.839	TRUE
cg16024485	OPRL1	NM_182647.1	36.1	20	62182186	0.807	TRUE
cg03896970	OPRL1	NM_182647.1	36.1	20	62182313	0.898	TRUE
cg24377504	OPRL1	NM_182647.1	36.1	20	62182449	0.889	FALSE
cg00314411	OPRL1	NM_182647.1	36.1	20	62182629	0.843	FALSE
cg12902246	RGS19	NM_005873.1	36.1	20	62182990	0.782	FALSE
cg18989937	RGS19	NM_005873.1	36.1	20	62183147	0.741	FALSE
cg20391608	COMT	NM_007310.1	36.1	22	18308581	0.8	FALSE
cg03446957	COMT	NM_007310.1	36.1	22	18309130	0.908	TRUE
cg22196019	COMT	NM_007310.1	36.1	22	18309697	0.92	FALSE
cg05989757	MAPK1	NM_002745.4	36.1	22	20551158	0.815	TRUE
cg17659879	MAPK1	NM_002745.4	36.1	22	20551906	0.931	TRUE
cg01196743	MAPK1	NM_002745.4	36.1	22	20552424	0.81	TRUE
cg22917359	MAPK1	NM_002745.4	36.1	22	20553228	0.878	FALSE
cg27580859	MAOA	NM_000240.2	36.1	X	43398803	0.813	TRUE
cg14009433	MAOA	NM_000240.2	36.1	X	43399450	0.889	TRUE
cg09356486	MAOA	NM_000240.2	36.1	X	43400106	0.906	FALSE
cg09883163	MAOA	NM_000240.2	36.1	X	43400508	0.893	TRUE
cg12338263	MAOB	NM_000898.3	36.1	X	43626077	0.945	FALSE
cg20550533	MAOB	NM_000898.3	36.1	X	43626471	0.841	TRUE
cg05876300	MAOB	NM_000898.3	36.1	X	43626728	0.88	TRUE
cg26000542	MAOB	NM_000898.3	36.1	X	43626834	0.816	TRUE
cg24323887	HTR2C	NM_000868.1	36.1	X	113722831	0.855	FALSE
cg17636534	HTR2C	NM_000868.1	36.1	X	113723566	0.916	TRUE
cg19491443	HTR2C	NM_000868.1	36.1	X	113723917	0.842	TRUE
cg23623863	HTR2C	NM_000868.1	36.1	X	113724321	0.892	FALSE
cg05010363	HTR2C	NM_000868.1	36.1	X	113724729	0.844	TRUE
cg02156408	HTR2C	NM_000868.1	36.1	X	113725033	0.877	TRUE
cg06841599	HTR2C	NM_000868.1	36.1	X	113725322	0.849	TRUE
cg06840457	HTR2C	NM_000868.1	36.1	X	113725764	0.914	TRUE
cg04055210	GABRA3	NM_000808.2	36.1	X	151370101	0.916	FALSE
cg04941592	MECP2	NM_004992.2	36.1	X	153016721	0.848	TRUE

cg11722044	MECP2	NM_004992.2	36.1	X	153016992	0.805	FALSE
cg05408953	MECP2	NM_004992.2	36.1	X	153017705	0.877	FALSE
cg05227791	MECP2	NM_004992.2	36.1	X	153017847	0.855	TRUE

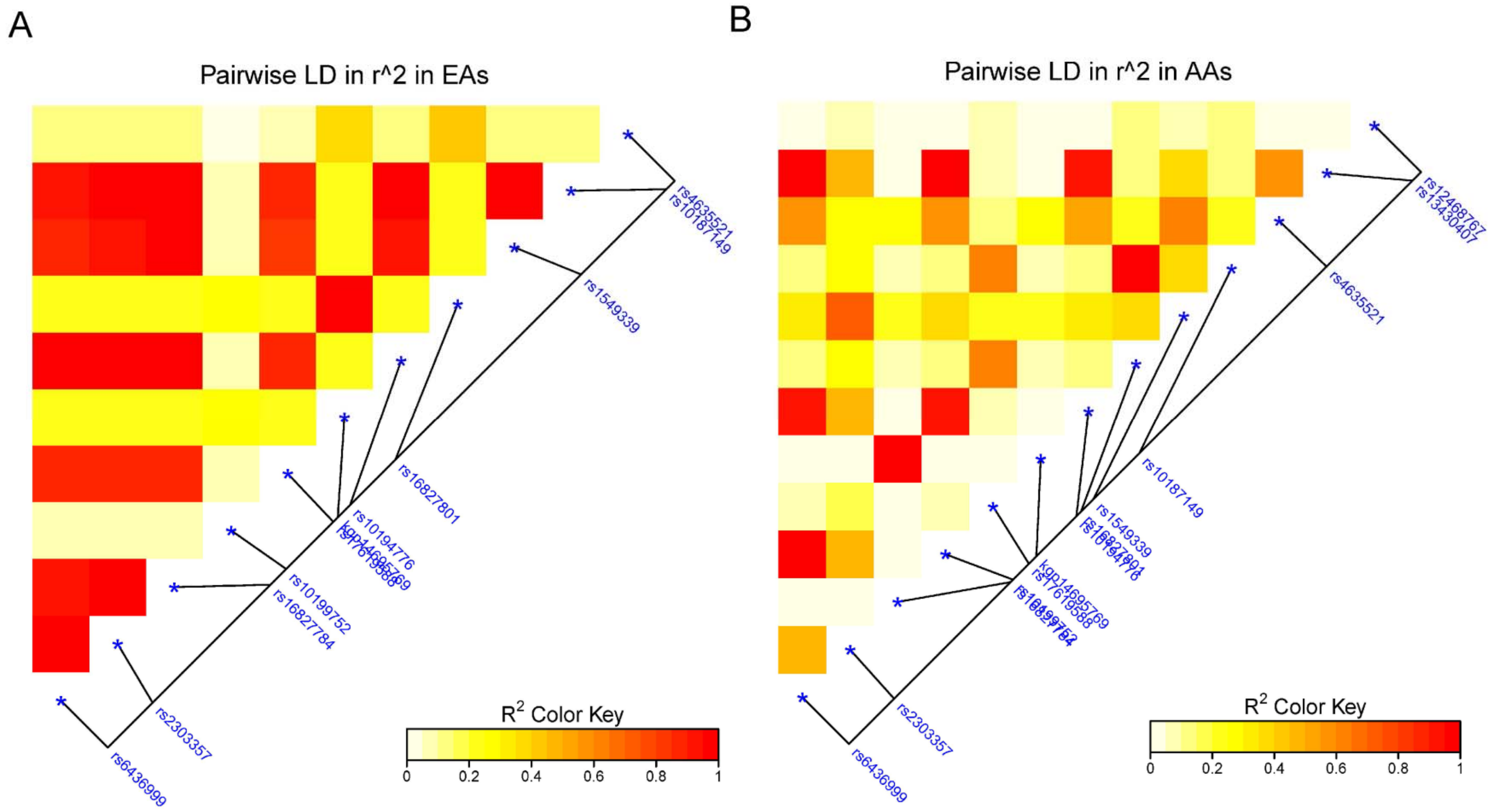

Figure S1. Linkage disequilibrium (LD) plots of 13 SNPs near CpG cg27531267 in gene *HTR2B*

The color of the diamond indicates the degree of pair-rise relationships among 13 SNPs, from low (white color) to high (red color).
